# Supplementary material for: Monitoring of the Pre-Equilibrium Step in the Alkyne Hydration Reaction Catalyzed by Au(III) Complexes: A Computational Study Based on Experimental Evidences
Source: Molecules. 2021 Apr 22;26(9):2445. doi: 10.3390/molecules26092445 (PMC8122707; doi:10.3390/molecules26092445)
Supplement: Supplementary file 1 [file molecules-26-02445-s001.zip › molecules-1183656-supplementary.pdf]

Supplementary Materials

Monitoring of the Pre-Equilibrium Step in the Alkyne Hydration Reaction Catalyzed by Au(III) Complexes: A Computational Study Based on Experimental Evidences

Flavio Sabatelli [1], Jacopo Segato [2], Leonardo Belpassi [1,3], Alessandro Del Zotto [2], Daniele Zuccaccia [2,*] and Paola Belanzoni [1,3,*]

[1] Dipartimento di Chimica, Biologia e Biotecnologie, Università degli Studi di Perugia, Via Elce di Sotto 8, I-06123, Perugia, flavio.sabatelli@gmail.com
[2] Dipartimento di Scienze Agroalimentari, Ambientali e Animali, Sezione di Chimica, Università di Udine, Via Cotonificio 108, I-33100 Udine, Italy; segato.jacopo@spes.uniud.it (J.S.); alessandro.delzotto@uniud.it (A.D.Z.)
[3] Istituto di Scienze e Tecnologie Chimiche del CNR "Giulio Natta" (CNR-SCITEC); leonardo.belpassi@cnr.it
* Correspondence: daniele.zuccaccia@uniud.it (D.Z.); paola.belanzoni@unipg.it (P.B.)

Contents

Experimental study – General procedures and materials - ^{1}H-NMR spectra…...................S2

Computational study – Figure S12……...S11

Coordination ability – comparative study between $[(ppy)Au(IPr)]^{2+}$, model $[(ppy)Au(NHC)]^{2+}$ and $[(NHC)Au]^{+}$ fragments……...S12

Figure S16…………………….…………………………………………….……….S22

Table S2………………………….……………………………………………..……….S23

Methodological study – explicit evaluation of dispersion and solvent effects on bonding energies and geometries of $[(ppy)Au(IPr)X]^{+/2+}$ and $[(ppy)Au(NHC)X]^{+/2+}$ (X = Cl^{-}, BF_4^{-}, OTf^{-}, H_2O, 2-butyne, 3-hexyne) complexes……………………………………..………………………S24

References……………………………………………………………………………S41

Experimental study - General procedures and materials

$NaAuCl_4 \cdot H_2O$, phenylpyridine, 3-hexyne, γ-valerolactone, silver triflate (AgOTf), silver tetrafluoroborate ($AgBF_4$), and solvents were purchased from Sigma Aldrich. All the solvents were used as received without any further purification, unless otherwise stated. [(ppy)Au(IPr)Cl]Cl was synthesized according to the literature.[1,2]

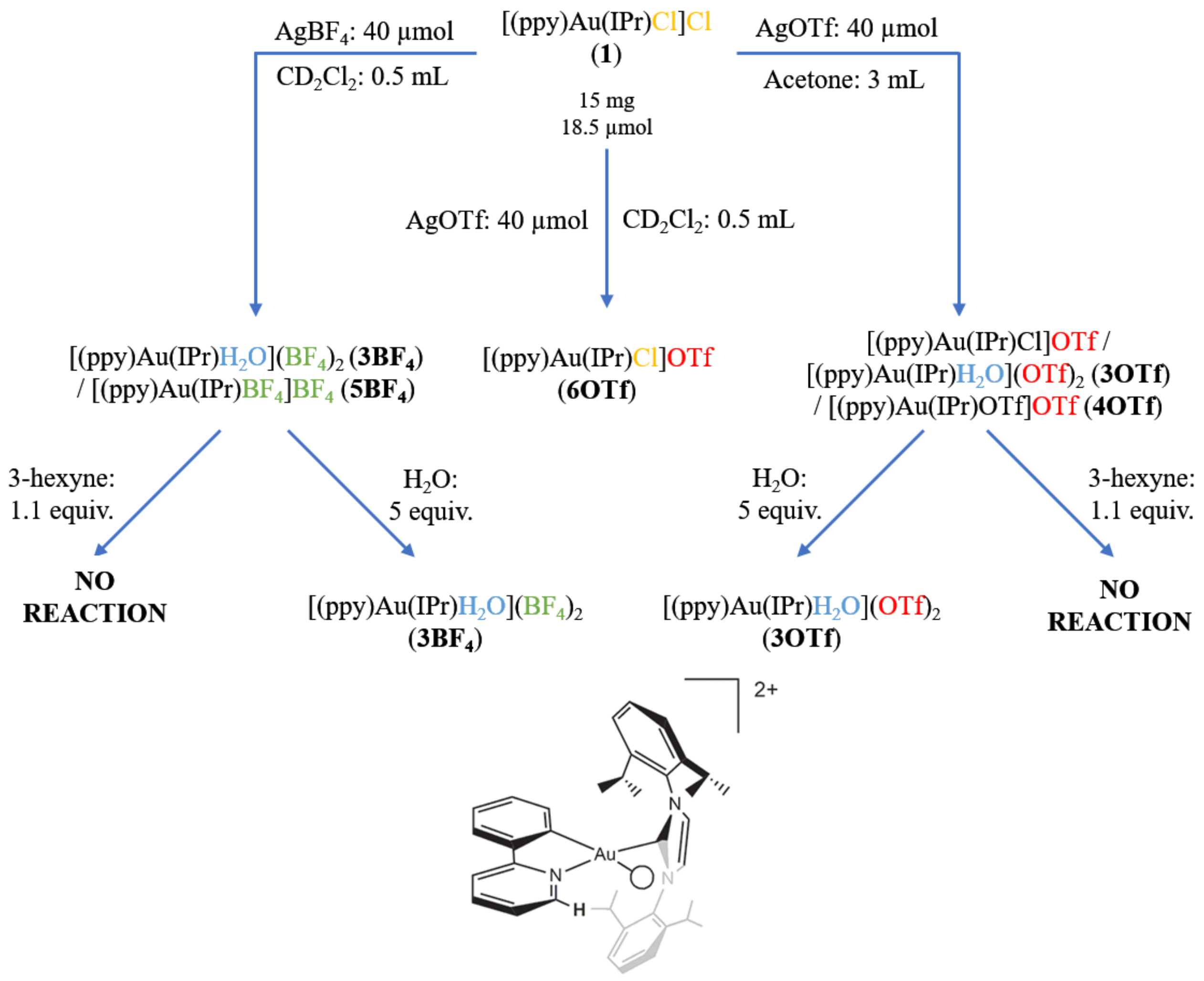

Scheme S1. List of the reactions studied in this work.

[(ppy)Au(IPr)BF₄]BF₄ (5BF₄)

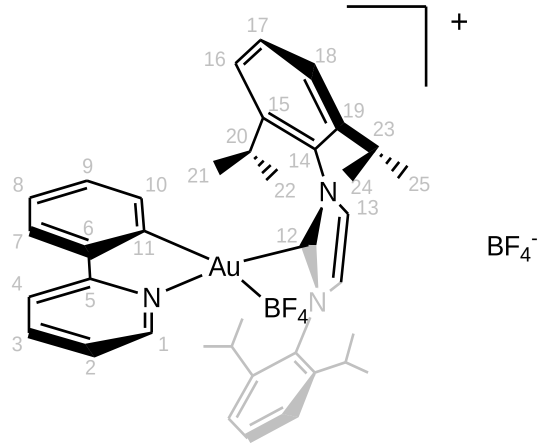

Figure S1. **5BF4** species

[(ppy)Au(IPr)BF_4]BF_4 was generated in a NMR tube by the reaction between [(ppy)Au(IPr)Cl]Cl (15 mg, 18.5 μmol) and 2 equiv (40 μmol) of $AgBF_4$ in 0.5 mL of CD_2Cl_2. The water complex [(ppy)Au(IPr)H_2O]$(BF_4)_2$ (48%) is also present in solution.

^{1}H NMR (400 MHz, CD_2Cl_2, 298 K): δ(ppm) = 8.55 (br, H1), 8.16 (br), 7.92 (d), 7.85-7.20 (m), 6.73 (d, 1H, $^3J_{HH}$ = 7.4 Hz, H10), 2.95 (br, H20), 2.65 (br, H23), 1.48 (H24), 1.30 (H22), 1.15 (d, $^3J_{HH}$ = 6.6 Hz, H25), 0.84 (H21).

^{19}F NMR (376 MHz, CD_2Cl_2, 298 K): δ(ppm) = -152.90 (d, J = 20.8 Hz).

[(ppy)Au(IPr)H₂O](BF₄)₂ (3BF₄)

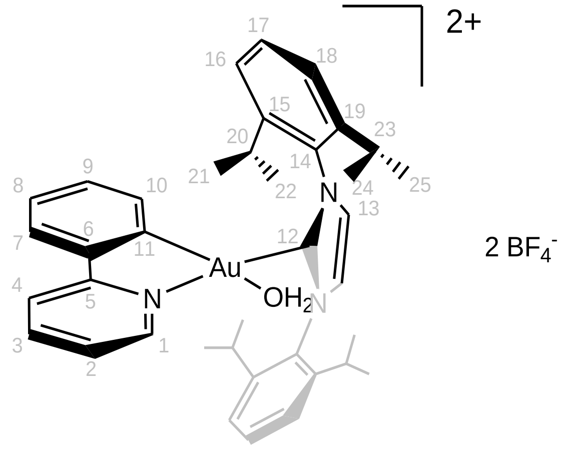

Figure S2. **3BF4** species

To the previous solution, 5 equiv of water was added giving the desired product. The complex was characterized by mono and bidimensional ^{1}H, ^{13}C and ^{19}F NMR experiments. The assignment of all ^{1}H, ^{13}C was made with the help of bidimensional experiments, such as ^{1}H-^{1}H COSY, ^{1}H-^{13}C HSQC, ^{1}H-^{13}C HMBC, and ^{1}H-^{1}H NOESY.

^{1}H NMR (400 MHz, CD_2Cl_2, 298 K): δ(ppm) = 8.75 (dt, 1H, $^{3}J_{HH}$ = 5.9, $^{4}J_{HH}$ = 2.0 Hz, H1), 8.18 (td, 1H, $^{3}J_{HH}$ = 7.9, $^{4}J_{HH}$ = 1.6 Hz, H3), 7.93 (dt, 1H, $^{3}J_{HH}$ = 8.2, $^{4}J_{HH}$ = 1.1 Hz, H4), 7.71 (s, 2H, H13), 7.66 (dt, 1H, $^{3}J_{HH}$ = 7.8, $^{4}J_{HH}$ = 1.8 Hz, H7), 7.61 – 7.52 (m, 3H, H2-17), 7.48 - 7.41 (m, 3H, H8-18), 7.32 (dd, 2H, $^{3}J_{HH}$ = 7.8, $^{4}J_{HH}$ = 1.5 Hz, H16), 7.25 (tt, 1H, $^{3}J_{HH}$ = 7.7, $^{4}J_{HH}$ = 1.8 Hz, H9), 6.98 (ddd, 1H, $^{3}J_{HH}$ = 7.9, $^{4}J_{HH}$ = 3.5 Hz, H10), 2.94 (m, 4H, H20-23), 1.46 (d, 6H, $^{3}J_{HH}$ = 6.6 Hz, H24), 1.33 (d, 6H, $^{3}J_{HH}$ = 6.7 Hz, H22), 1.13 (d, 6H, $^{3}J_{HH}$ = 6.8 Hz, H25), 0.94 (d, 6H, $^{3}J_{HH}$ = 6.8 Hz, H21)

^{13}C {^{1}H} NMR (101 MHz, CD_2Cl_2, 298 K) δ(ppm) = 162.20 (1C, C5), 150.56 (1C, C12), 147.36 (1C, C19), 145.61 (d, 1C, C1), 144.91 (1C, C15), 144.16 (1C, C3), 143.05 (1C, C11), 134.70 (1C, C6), 133.32 (d, 1C, C10), 132.73 (1C, C9), 132.12 (2C, C14), 131.83(2C, C17), 129.52 (1C, C8), 127.14 (2C, C13), 126.47 (1C, C7), 124.99-124.96 (4C, C16-18), 124.42 (1C, C2), 120.97 (1C, C4), 29.22-29.17 (4C, C20-23), 26.31 (2C, C22), 26.18 (2C, C25), 22.68 (2C, C21), 22.16-22.10 (d, 2C, C24).

^{19}F NMR (376 MHz, CD_2Cl_2, 298 K): δ(ppm) = -152.74 (d, J = 20.8 Hz).

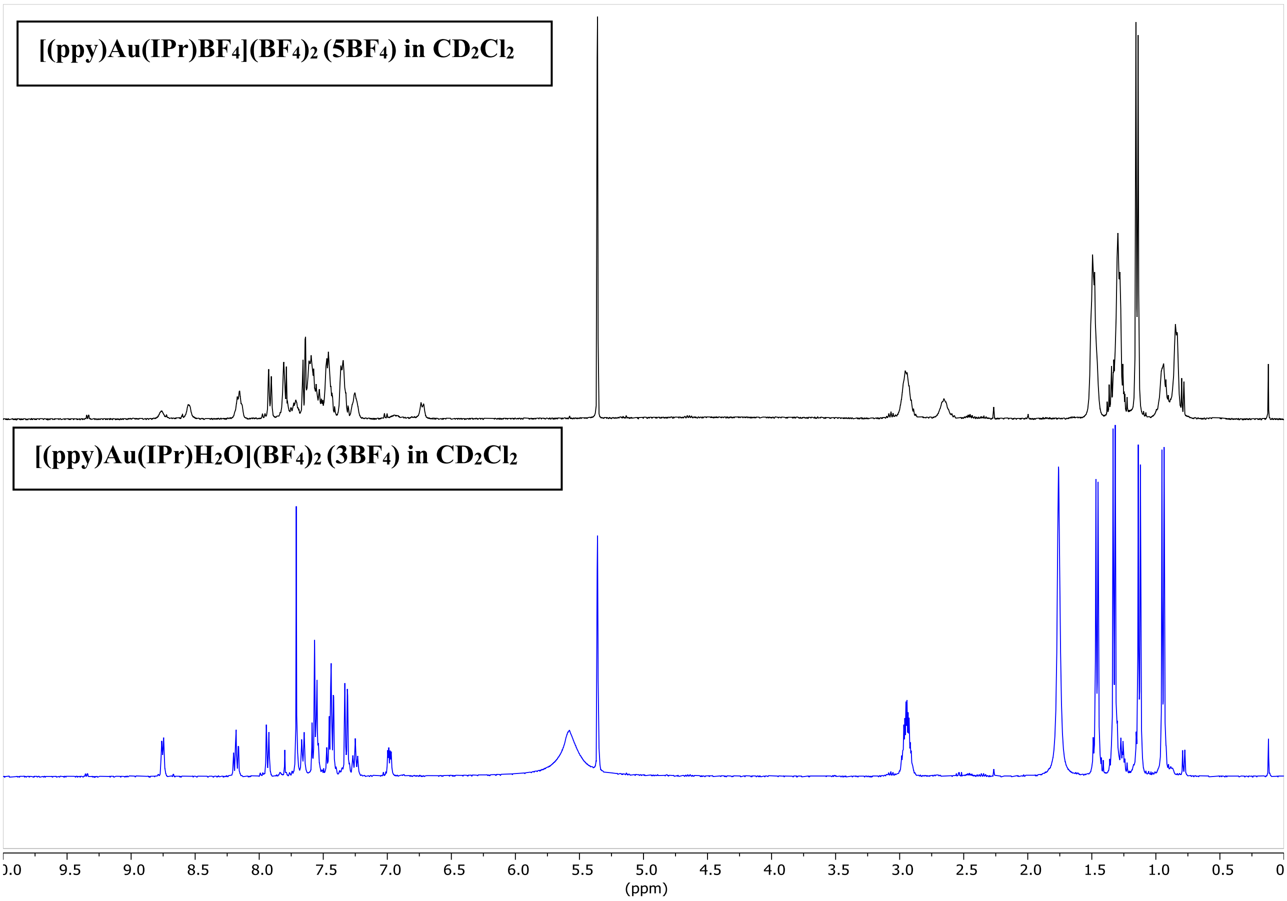

Figure S3. ^{1}H-NMR spectra in CD_2Cl_2 of **5BF4** (top) and **3BF4** (bottom) complexes

[(ppy)Au(IPr)Cl]OTf (6OTf)

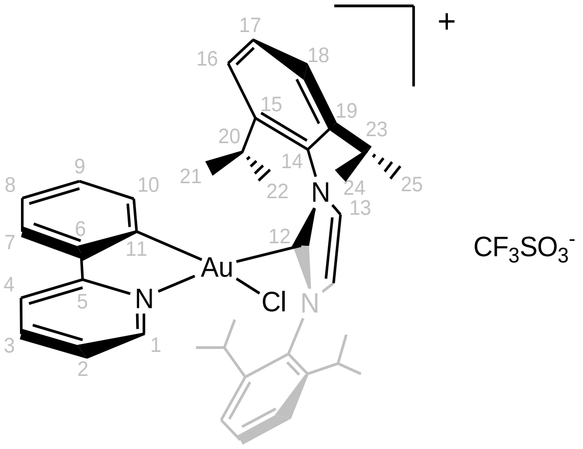

Figure S4. **6OTf** species

The complex [(ppy)Au(IPr)Cl]OTf was synthesized by adding 20 µmol of silver triflate (1.1 equiv) to 15 mg (18.5 µmol) of [(ppy)Au(IPr)Cl]Cl in 5 mL of dichloromethane. The solution was filtered on Celite and the volume of the solution was then reduced. The desired compound was precipitated by addition of pentane. The complex was characterized by mono and bidimensional ^{1}H, ^{13}C, and ^{19}F NMR experiments. The assignment of all ^{1}H, ^{13}C was made with the help of bidimensional experiments, such as ^{1}H-^{1}H COSY, ^{1}H-^{13}C HSQC, ^{1}H-^{13}C HMBC, and ^{1}H-^{1}H NOESY.

^{1}H NMR(400 MHz, $CDCl_3$, 298 K): δ(ppm) = 9.27 (dd, 1H, $^{3}J_{HH}$ = 6.1, $^{4}J_{HH}$ = 1.5 Hz, H1), 8.21 (td, 1H, $^{3}J_{HH}$ = 7.8 Hz, $^{4}J_{HH}$ = 1.6 Hz, H3), 8.11 (dd, 1H, $^{3}J_{HH}$ = 8.3, $^{4}J_{HH}$ =1.6 Hz, H4), 7.88 (s, 2H, H13), 7.78 (dd, 1H, $^{3}J_{HH}$ = 7.9 Hz, $^{4}J_{HH}$ = 1.6 Hz, H7), 7.53 – 7.40 (m, 4H, H2-8-17), 7.38 – 7.29 (m, 3H, H9-18), 7.22 (dd, 2H, $^{3}J_{HH}$ = 7.8, $^{4}J_{HH}$ = 1.5 Hz, H16), 6.96 (d, 1H, $^{3}J_{HH}$ = 7.8 Hz, H10), 3.03 (hept, 2H, $^{3}J_{HH}$ = 6.7 Hz, H20), 2.86 (hept, 2H, $^{3}J_{HH}$ = 6.7 Hz, H23), 1.43 (d, 6H, $^{3}J_{HH}$ = 6.6 Hz, H24), 1.23 (d, 6H, $^{3}J_{HH}$ = 6.7 Hz, H22), 1.09 (d, 6H, $^{3}J_{HH}$ = 6.8 Hz, H25), 0.70 (d, 6H, $^{3}J_{HH}$ = 6.7 Hz, H21).

^{13}C{^{1}H} NMR (101 MHz, $CDCl_3$, 298 K) δ = 163.79 (s, 1C, C5), 148.67(s, 1C, C12), 148.53 (s, 1C, C6), 147.40 (s, 1C, C1), 147.21 (s, 2C, C19), 145.04 (s, 2C, C15), 144.14 (s, 1C, C3), 143.19 (s, 1C, C11), 132.89 (s, 1C, C9), 132.53 (s, 1C, C14), 132.47 (s, 1C, C10), 131.91 (s, 2C, C17), 129.87 (s, 1C, C8), 128.66 (s, 2C, C13), 126.96 (s, 1C, C7), 125.13 (s, 2C, C16), 124.83 (s, 2C, C18), 124.56

(s, 1C, C2), 121.88 (s, 1C, C4), 29.49 (s, 2C, C23), 29.06 (s, 2C, C20), 26.79 (s, 2C, C25), 26.56 (s, 2C, C22), 22.80 (s, 2C, C21), 22.66 (s, 2C, C24).
^{19}F NMR (376 MHz, $CDCl_3$, 298 K): δ(ppm) = −78.08.

[(ppy)Au(IPr)OTf]OTf (4OTf)

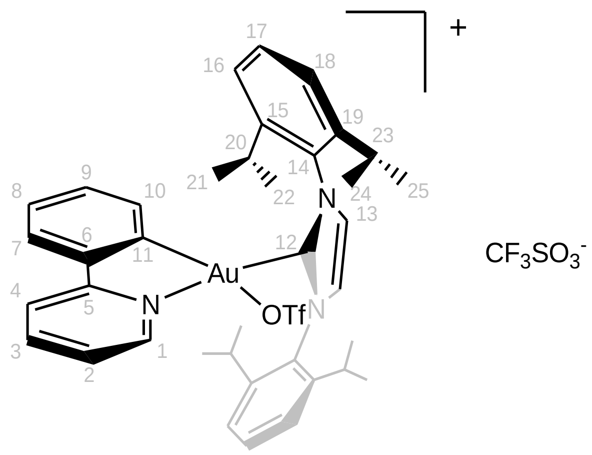

Figure S5. **4OTf** species

The complex [(ppy)Au(IPr)OTf]OTf was synthesized by adding 2.2 equiv of silver triflate to 15 mg (18.5 µmol) of [(ppy)Au(IPr)Cl]Cl dissolved in 3 mL of acetone. The solution was filtered on Celite and the solvent was then removed by vacuum. The solid was dissolved in dichloromethane and the desired compound was precipitated by adding pentane. The complex was characterized by mono and bidimensional ^{1}H and ^{19}F NMR experiments. The assignment of all ^{1}H was made by comparison with the resonances of the starting complex.

^{1}H NMR (400 MHz, CD_2Cl_2, 298 K): δ (ppm)= 8.79 (d, 1H, $^{3}J_{HH}$ = 5.9 Hz, H1), 8.19 (t, 1H, $^{3}J_{HH}$ = 7.8 Hz, H3), 7.92 (d, 1H, $^{3}J_{HH}$ = 8.1 Hz,H4), 7.79 (s, 2H, H13), 7.71 (d, 1H, $^{3}J_{HH}$ = 7.79 Hz, H7), 7.67 – 7.59 (m, 2H, H17), 7.57 – 7.46 (m, 3H, H2-18), 7.36 (m, 3H, H8-16), 7.26 (td, 1H, $^{3}J_{HH}$ = 7.8, $^{4}J_{HH}$ = 1.7 Hz, H9), 6.72 (d, 1H, $^{3}J_{HH}$ = 8.1 Hz, H10), 2.96 (p, 2H, $^{3}J_{HH}$ = 6.7 Hz, H20), 2.66 (p, 2H, $^{3}J_{HH}$ = 6.7 Hz, H23), 1.52 (d, 7H, $^{3}J_{HH}$ = 6.4 Hz, H24), 1.29 (d, 8H, $^{3}J_{HH}$ = 6.4 Hz, H22), 1.15 (d, 8H, $^{3}J_{HH}$ = 6.5 Hz, H25), 0.85 (d, 6H, $^{3}J_{HH}$ = 6.7 Hz, H21).
^{19}F NMR (376 MHz, CD_2Cl_2, 298 K): δ(ppm) = −77.95.

[(ppy)Au(IPr)H_2O]$(OTf)_2$ (3OTf)

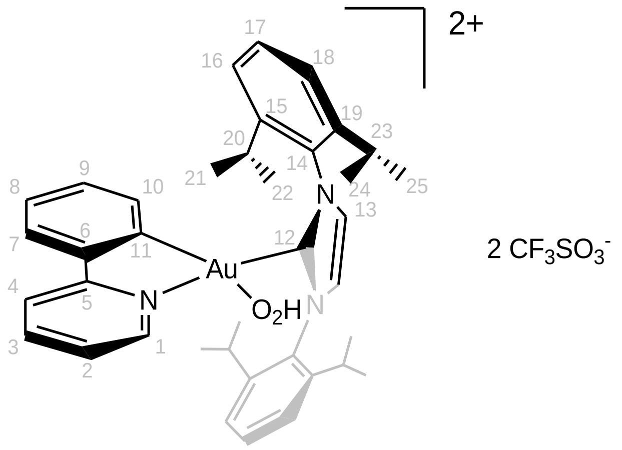

Figure S6. **3OTf** species

To a dichloromethane solution of complex **5OTf**, was added 5 equiv of water was added, giving the desired product. The complex was characterized by mono and bidimensional ^{1}H, ^{13}C, and ^{19}F NMR experiments. The assignment of all ^{1}H, ^{13}C was made with the help of bidimensional experiments such as ^{1}H-^{1}H COSY, ^{1}H-^{13}C HSQC, ^{1}H-^{13}C HMBC, and ^{1}H-^{1}H NOESY.

^{1}H NMR (400 MHz, CD_2Cl_2, 298 K): δ(ppm) = 8.75 (dt, 1H, $^{3}J_{HH}$ = 5.9, $^{4}J_{HH}$ = 2.0 Hz, H1), 8.18 (td, 1H, $^{3}J_{HH}$ = 7.9, $^{4}J_{HH}$ = 1.6 Hz, H3), 7.93 (dt, 1H, $^{3}J_{HH}$ = 8.2, $^{4}J_{HH}$ = 1.1 Hz, H4), 7.71 (s, 2H, H13), 7.66 (dt, 1H, $^{3}J_{HH}$ = 7.8, $^{4}J_{HH}$ = 1.8 Hz, H7), 7.61 – 7.52 (m, 3H, H2-17), 7.48 - 7.41 (m, 3H, H8-18), 7.32 (dd, 2H, $^{3}J_{HH}$ = 7.8, $^{4}J_{HH}$ = 1.5 Hz, H16), 7.25 (tt, 1H, $^{3}J_{HH}$ = 7.7, $^{4}J_{HH}$ = 1.8 Hz, H9), 6.98 (ddd, 1H, $^{3}J_{HH}$ = 7.9, $^{4}J_{HH}$ = 3.5 Hz, H10), 2.94 (m, 4H, H20-23), 1.46 (d, 6H, $^{3}J_{HH}$ = 6.6 Hz, H24), 1.33 (d, 6H, $^{3}J_{HH}$ = 6.7 Hz, H22), 1.13 (d, 6H, $^{3}J_{HH}$ = 6.8 Hz, H25), 0.94 (d, 6H, $^{3}J_{HH}$ = 6.8 Hz, H21).

^{19}F NMR (376 MHz, CD_2Cl_2, 298 K): δ(ppm) = −78.08.

5BF4

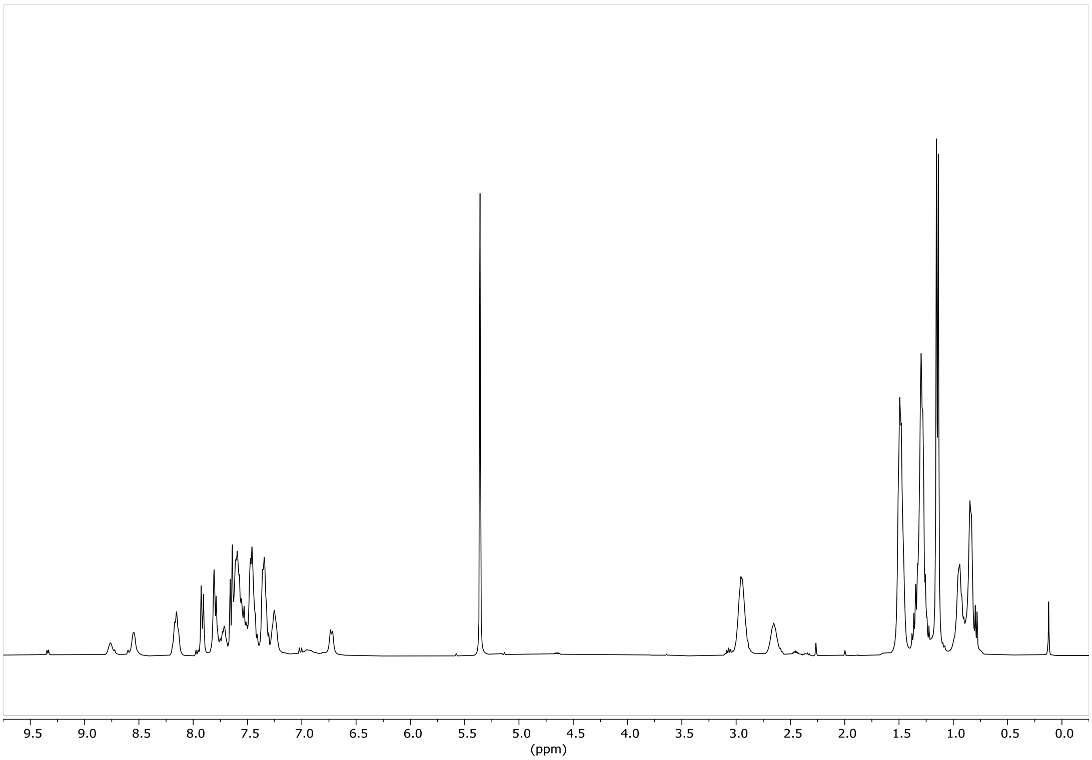

Figure S7: ^{1}H-NMR spectrum in CD_2Cl_2 of **5BF4** complex in presence of **3BF4**.

3BF4

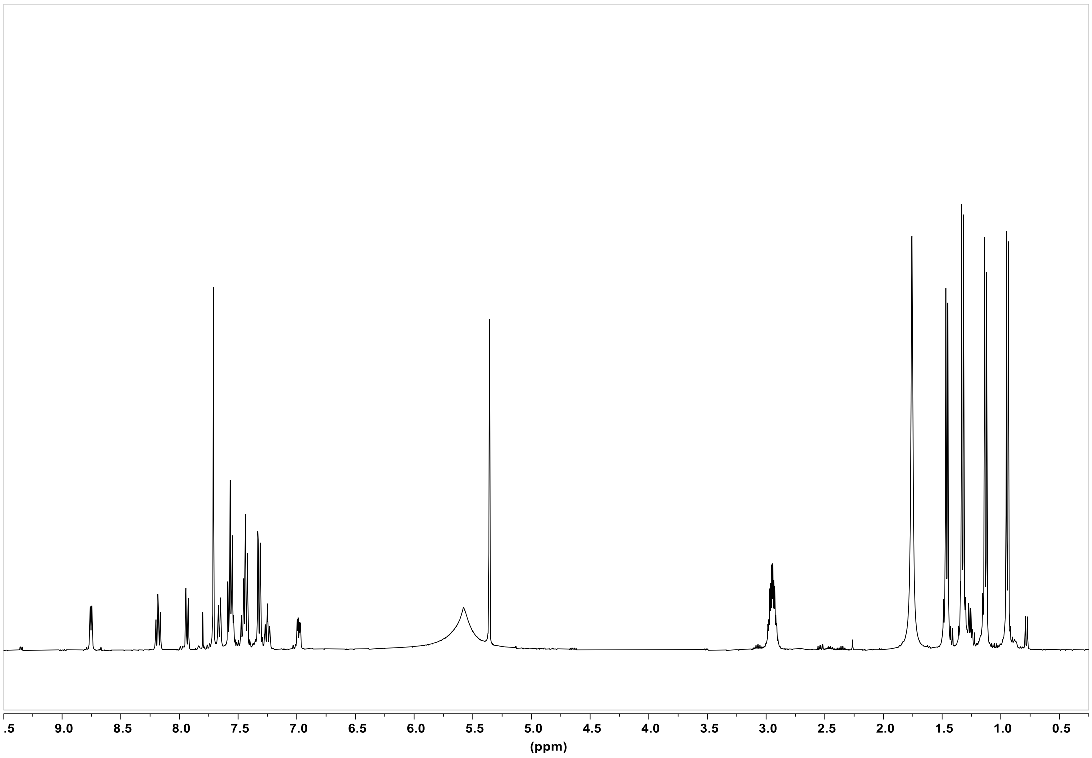

Figure S8: ^{1}H-NMR spectrum in CD_2Cl_2 of **$3BF_4$** complex in presence of excess of water.

6OTf

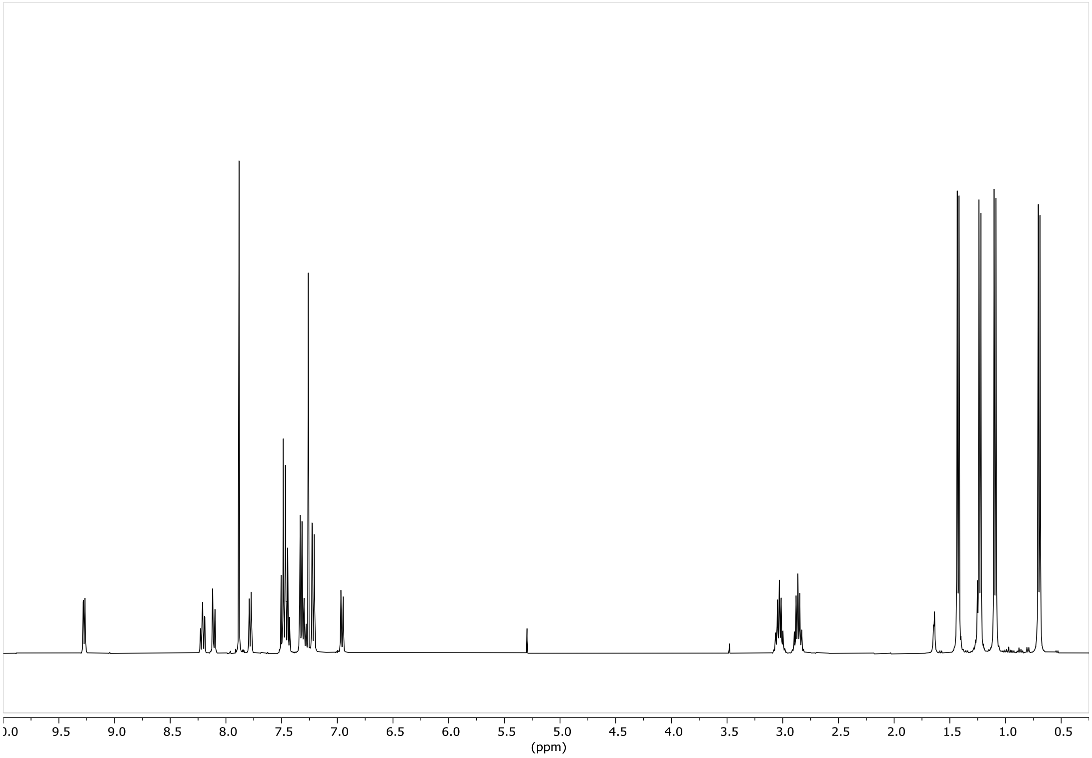

Figure S9: ^{1}H-NMR spectrum in CD_2Cl_2 of **6OTf** complex.

4OTf

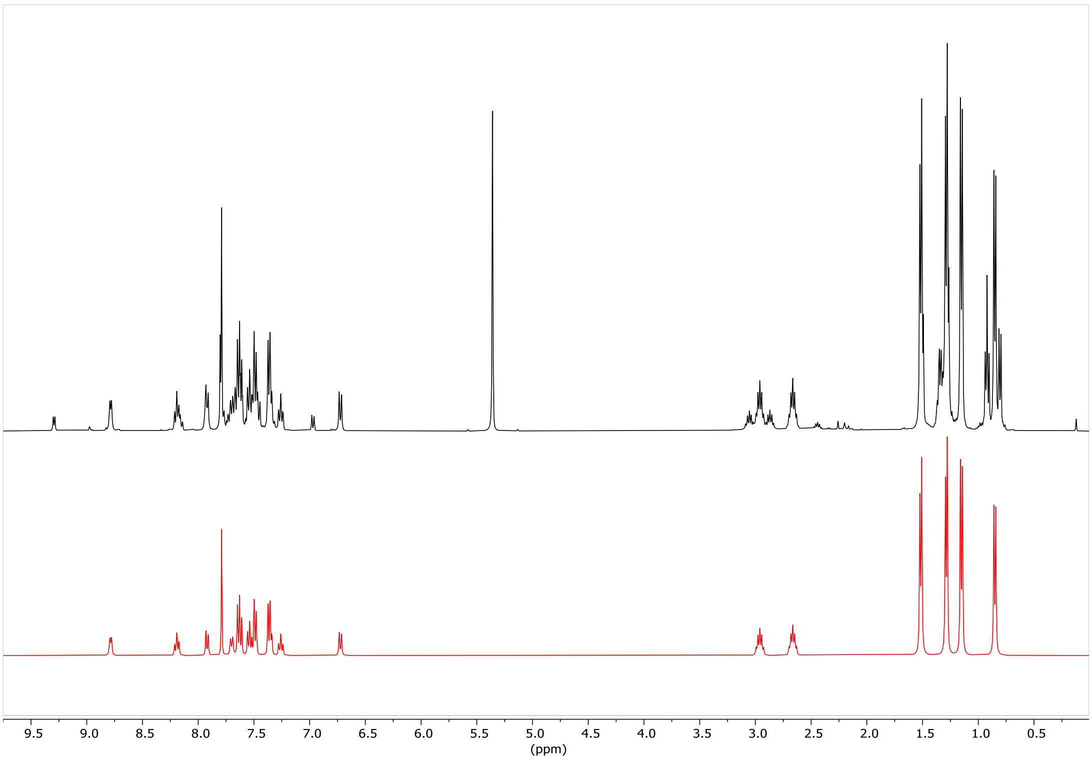

Figure S10: ^{1}H-NMR spectrum in CD_2Cl_2 of **4OTf** complex in the presence of [(ppy)Au(IPr)Cl]OTf. In red the deconvoluted spectra.

3OTf

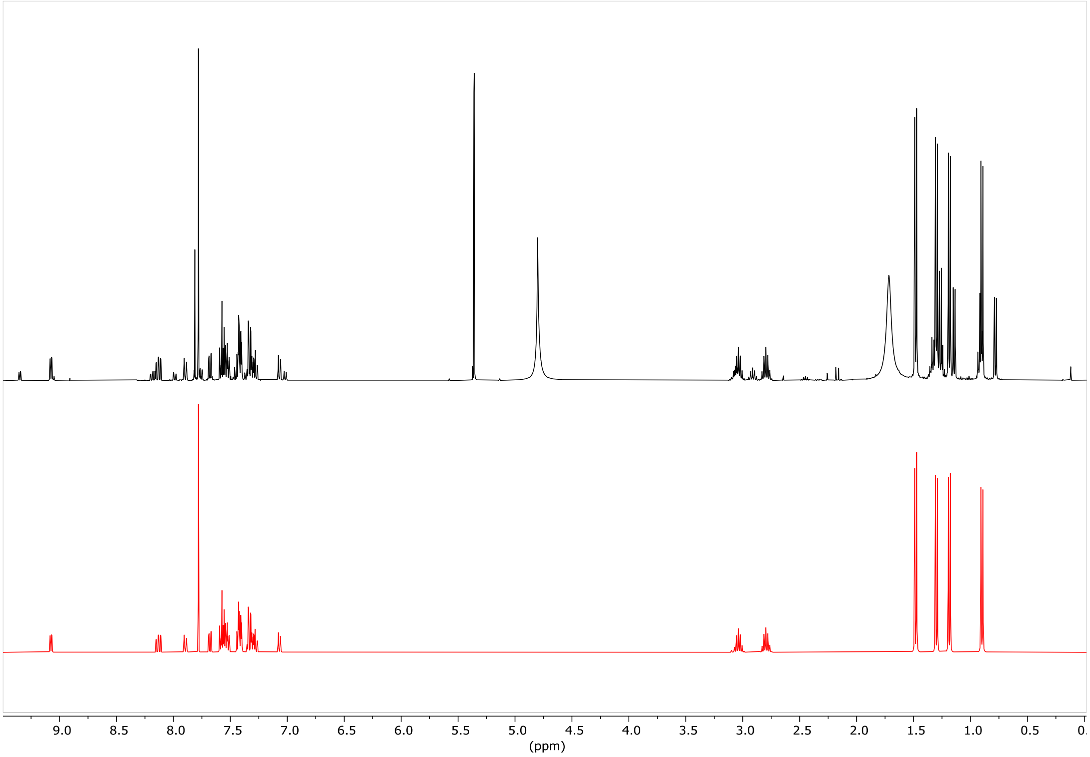

Figure S11: ^{1}H-NMR spectrum in CD_2Cl_2 of **3OTf** complex in the presence of [(ppy)Au(IPr)Cl]OTf. In red the deconvoluted spectra.

Computational study

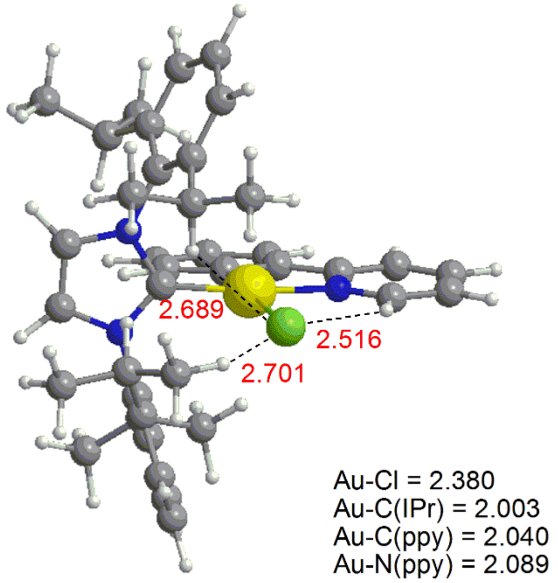

$[(ppy)Au(IPr)Cl]^{+}$

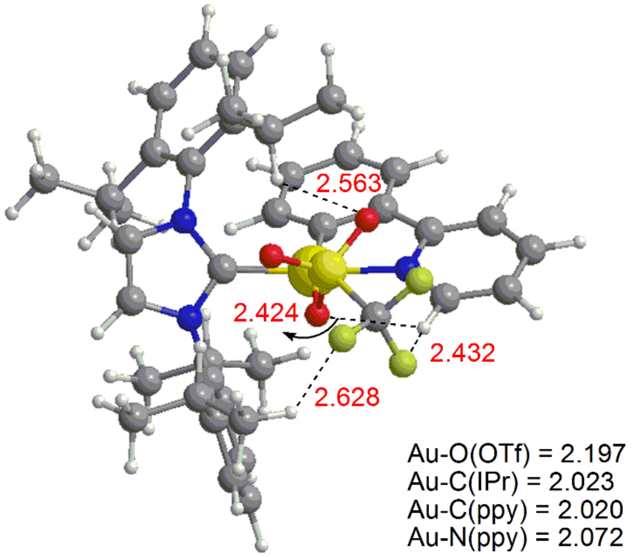

$[(ppy)Au(IPr)OTf]^{+}$

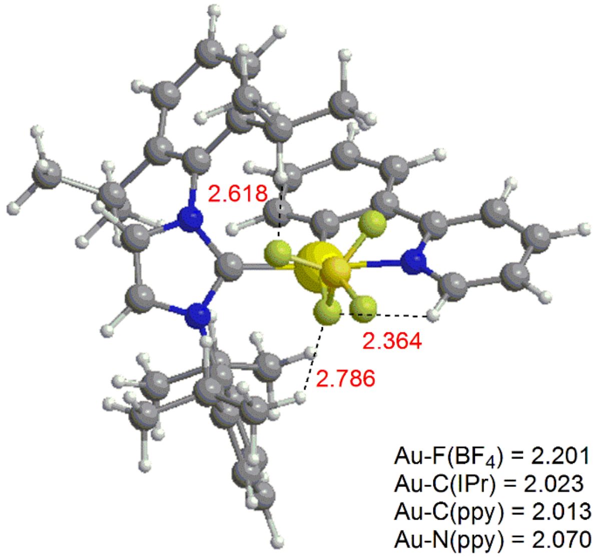

$[(ppy)Au(IPr)BF_4]^{+}$

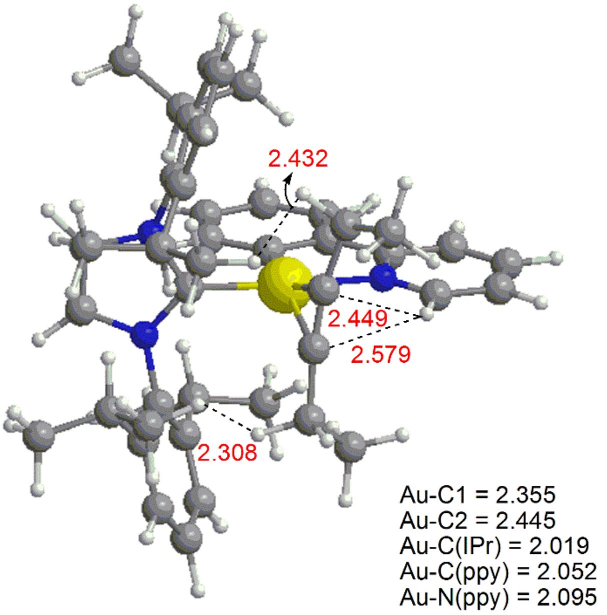

$[(ppy)Au(IPr)3\text{-hexyne}]^{2+}$

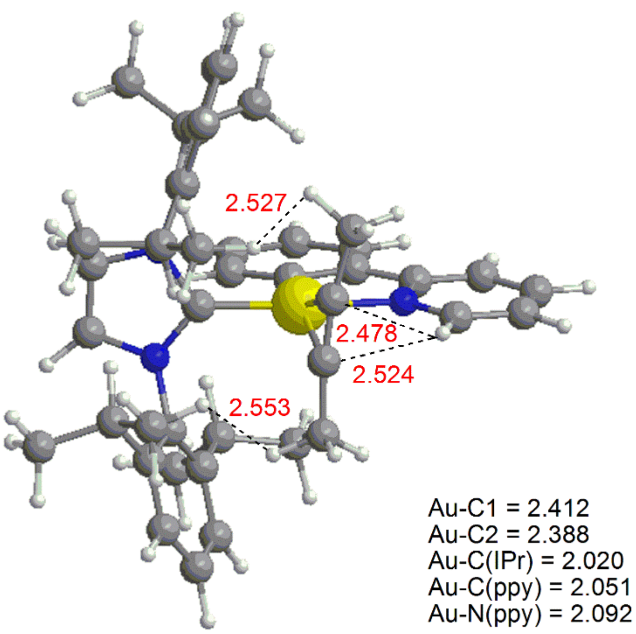

$[(ppy)Au(IPr)2\text{-butyne}]^{2+}$

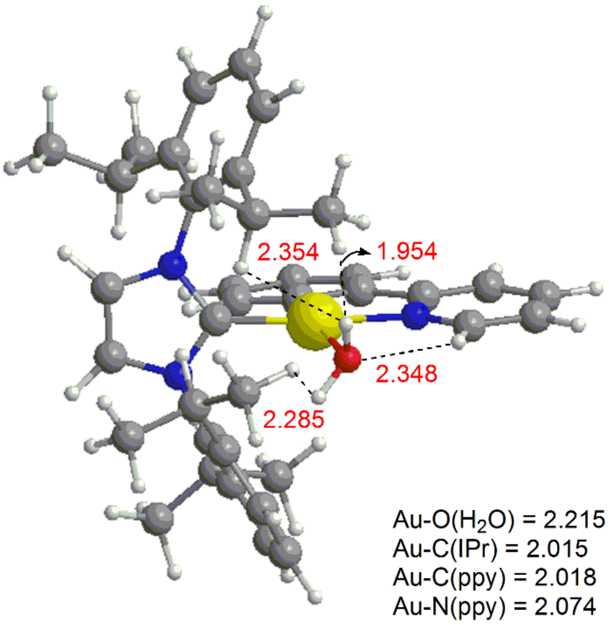

$[(ppy)Au(IPr)H_2O]^{2+}$

Figure S12: Optimized structures of $[(ppy)Au(IPr)X]^{+/2+}$ (X = Cl^-, BF_4^-, OTf^-, H_2O, 2-butyne, 3-hexyne) complexes. Main bond lengths are reported (Å). Principal interaction contacts (below 3 Å) of the IPr and ppy moieties with X ligands are highlighted in red color.

Coordination ability – comparative study between $[(ppy)Au(IPr)]^{2+}$, model $[(ppy)Au(NHC)]^{2+}$ and $[(NHC)Au]^{+}$ fragments

To compare the coordination ability of the $[(ppy)Au(IPr)]^{2+}$ fragment with that of the model $[(ppy)Au(NHC)]^{2+}$ (NHC = 1,3-dimethylimidazol-2-ylidene) and $[(NHC)Au]^{+}$ fragments, the bonding energies of X (X = Cl^-, BF_4^-, OTf^-, H_2O, 2-butyne and 3-hexyne) have been evaluated. We have performed calculations of free energy change (ΔG) for the following reactions:

$$[(ppy)Au(IPr)]^{2+} + X^{-/0} \rightarrow [(ppy)Au(IPr)X]^{+/2+} \qquad (1)$$

$$[(ppy)Au(NHC)]^{2+} + X^{-/0} \rightarrow [(ppy)Au(NHC)X]^{+/2+} \qquad (2)$$

$$[(NHC)Au]^{+} + X^{-/0} \rightarrow [(NHC)AuX]^{0/+} \qquad (3)$$

The results are summarized in Table S1.

	Cl^-	OTf^-	BF_4^-	3-hexyne	2-butyne	H_2O
$[(ppy)Au(III)(IPr)]^{2+}$						
ΔE	−54.3	−34.3	−24.7	−31.4	−27.4	−15.2
ΔH	−51.5	−32.4	−21.6	−27.5	−23.8	−13.4
ΔG	**−44.0**	**−17.5**	**−12.5**	**−15.9**	**−12.6**	**−0.4**
$[(ppy)Au(III)(NHC)]^{2+}$						
ΔE	−59.3	−45.1	−33.0	−33.3	−31.1	−21.9
ΔH	−56.3	−42.6	−30.6	−30.4	−28.4	−18.7
ΔG	**−48.8**	**−32.6**	**−22.6**	**−20.6**	**−19.6**	**−9.4**
$[(NHC)Au(I)]^{+}$						
ΔE	−54.4	−39.5	−28.2	−43.6	−41.4	−28.4
ΔH	−51.4	−38.8	−27.4	−42.1	−40.2	−27.0
ΔG	**−43.9**	**−27.5**	**−17.2**	**−31.3**	**−29.4**	**−16.4**

Table S1. X (X = Cl^-, BF_4^-, OTf^-, H2O, 3-hexyne, 2-butyne) bonding electronic energies ΔE, bonding enthalpies ΔH and bonding Gibbs free energies ΔG to $[(ppy)Au(III)(IPr)]^{2+}$, $[(ppy)Au(III)(NHC)]^{2+}$, and $[(NHC)Au(I)]^{+}$. Values are in kcal/mol.

The bonding Gibbs free energies span a range of −0.4/−44.0 kcal/mol for the experimental catalyst and of −9.4/−48.8 kcal/mol for the model one. For Au(I) complexes the bonding free energies are calculated between −16.4 and −43.9 kcal/mol. The overall ΔG and ΔE trends in all the different systems can be readily compared in Figure S13. The same qualitative trends can be found with ΔE and ΔG values.

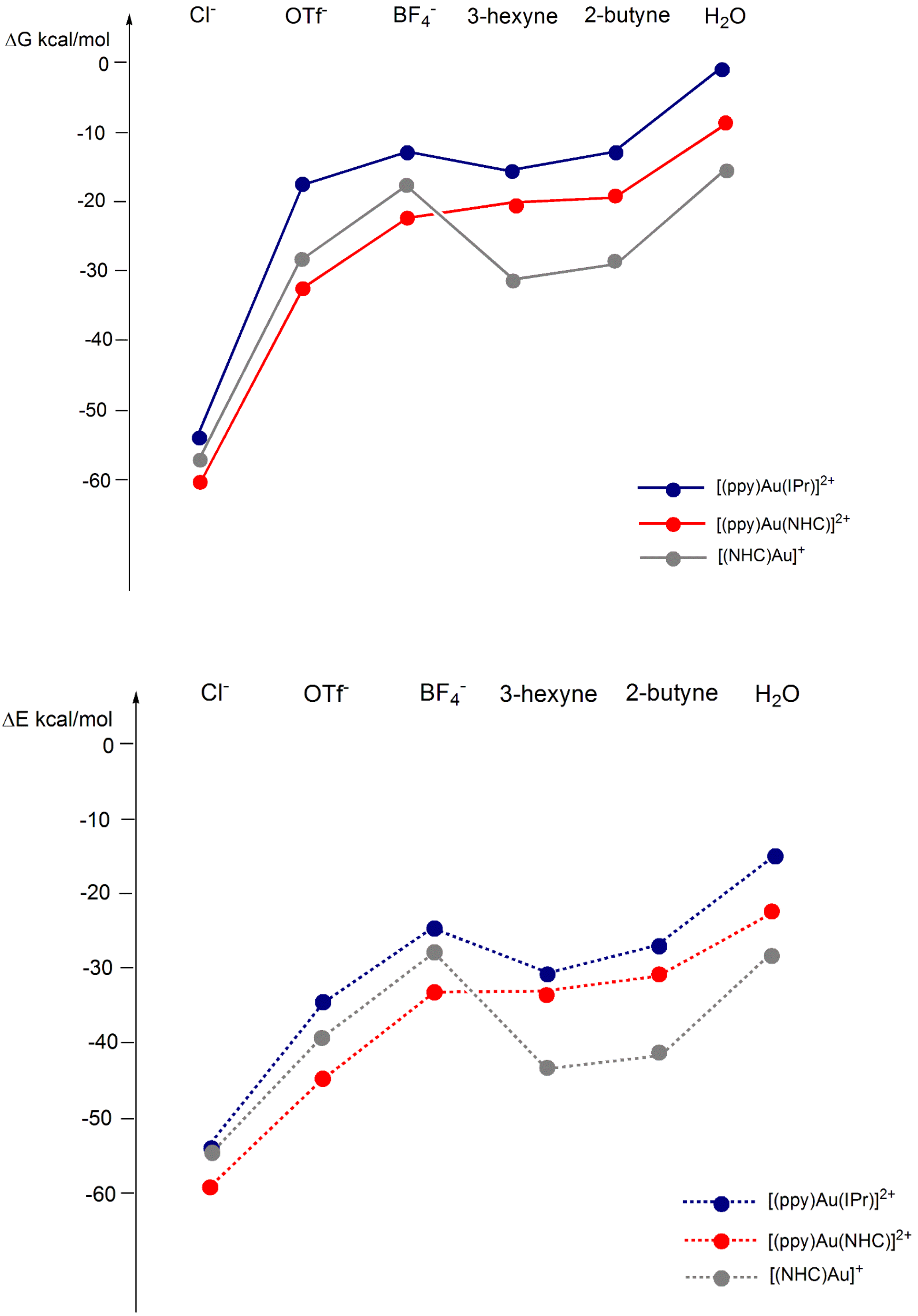

Figure S13. Bonding free energy ΔG (top) and electronic energy ΔE (bottom) trend for $[(ppy)Au(IPr)X]^{+/2+}$, $[(ppy)Au(NHC)X]^{+/2+}$, and $[(NHC)AuX]^{0/+}$ complexes (X = Cl^-, BF_4^-, OTf^-, H_2O, 2-butyne, 3-hexyne). Plotted data are taken from Table S1.

We can immediately see that the anionic ligands exhibit the largest bonding interactions compared to the neutral ones towards the model $[(ppy)Au(NHC)]^{2+}$fragment. In particular, Au(III)Cl shows the highest bonding energy, following the Au(III)Cl > Au(III)OTf > Au(III)BF_4 order, whereas bonding energies of neutral ligands are lower, with the minimum value calculated for H_2O, in the order Au(III)3-hexyne > Au(III)2-butyne > Au(III)H_2O. This energetic trend is not the same as that of $[(ppy)Au(IPr)X]^{+/2+}$ complexes (Cl^- > OTf^- > BF_4^- > 3-hexyne > 2-butyne > H_2O vs. Cl^- > OTf^- > 3-hexyne > 2-butyne ≈ BF_4^- > H_2O, respectively).

In general, the bonding energies along the Au(III) full complex series are always lower than the corresponding ones in the model Au(III) and Au(I) complex series. Interestingly, the $[(ppy)Au(NHC)X]^{+/2+}$ model systems, which should simulate the real ones, show a different stabilization depending on the X ligand. For instance, H_2O has a larger bonding energy in the $[(ppy)Au(NHC)X]^{2+}$ complex by 9.0 kcal/mol, whereas 2-butyne and 3-hexyne have larger ΔG by 7.0 and 4.7 kcal/mol, respectively, with respect to the $[(ppy)Au(IPr)X]^{2+}$ full complex. This difference is even more pronounced for the anionic X ligands: $[(ppy)Au(NHC)OTf]^{+}$ model complex is stabilized by 15.1 kcal/mol with respect to the real one, whereas $[(ppy)Au(NHC)BF_4]^{+}$ and $[(ppy)Au(NHC)Cl]^{+}$ model species are stabilized by 10.1 and 5.9 kcal/mol, respectively. This finding, showing a different energy gap between the model and the real systems, would suggest that replacing IPr by NHC could be not quantitatively safe for all the X ligands.

For the $[(NHC)AuX]^{0/+}$ complexes, the bonding ΔG follows the Au(I)Cl > Au(I)3-hexyne > Au(I)2-butyne> Au(I)OTf > Au(I)BF_4 > Au(I)H_2O trend, in agreement with previous results showing that 3-hexyne and OTf^- are more coordinating than BF_4^-.[3] Remarkably, neutral alkynes coordinate more strongly than anionic OTf^- and BF_4^- ligands. Although the difference in ΔG values between Au(III) model and Au(I) systems is approximately constant within the anionic ligand subset, it increases along the neutral ligand series moving from H_2O to 2-butyne and 3-hexyne. For instance, for the neutral X ligand series, the bonding free energies are larger for Au(I) than for Au(III) model by 7.0/10.7

kcal/mol, and for the anionic X they are larger for Au(III) model than for Au(I) by 3.3/5.4 kcal/mol. Thus, we can conclude that Au(III) interacts more strongly with anionic X than Au(I) does, as one should expect. Au(I) instead shows a larger bonding interaction with neutral X ligands.

The optimized structures of the $[(ppy)Au(NHC)X]^+$ and [(NHC)AuX] model complexes (X = Cl^-, BF_4^-, OTf^-, H_2O, 2-butyne, 3-hexyne) are shown in Figures S14 and S15, respectively, and they can be compared to those of the corresponding $[(ppy)Au(IPr)X]^+$ full complexes (Figure S12).

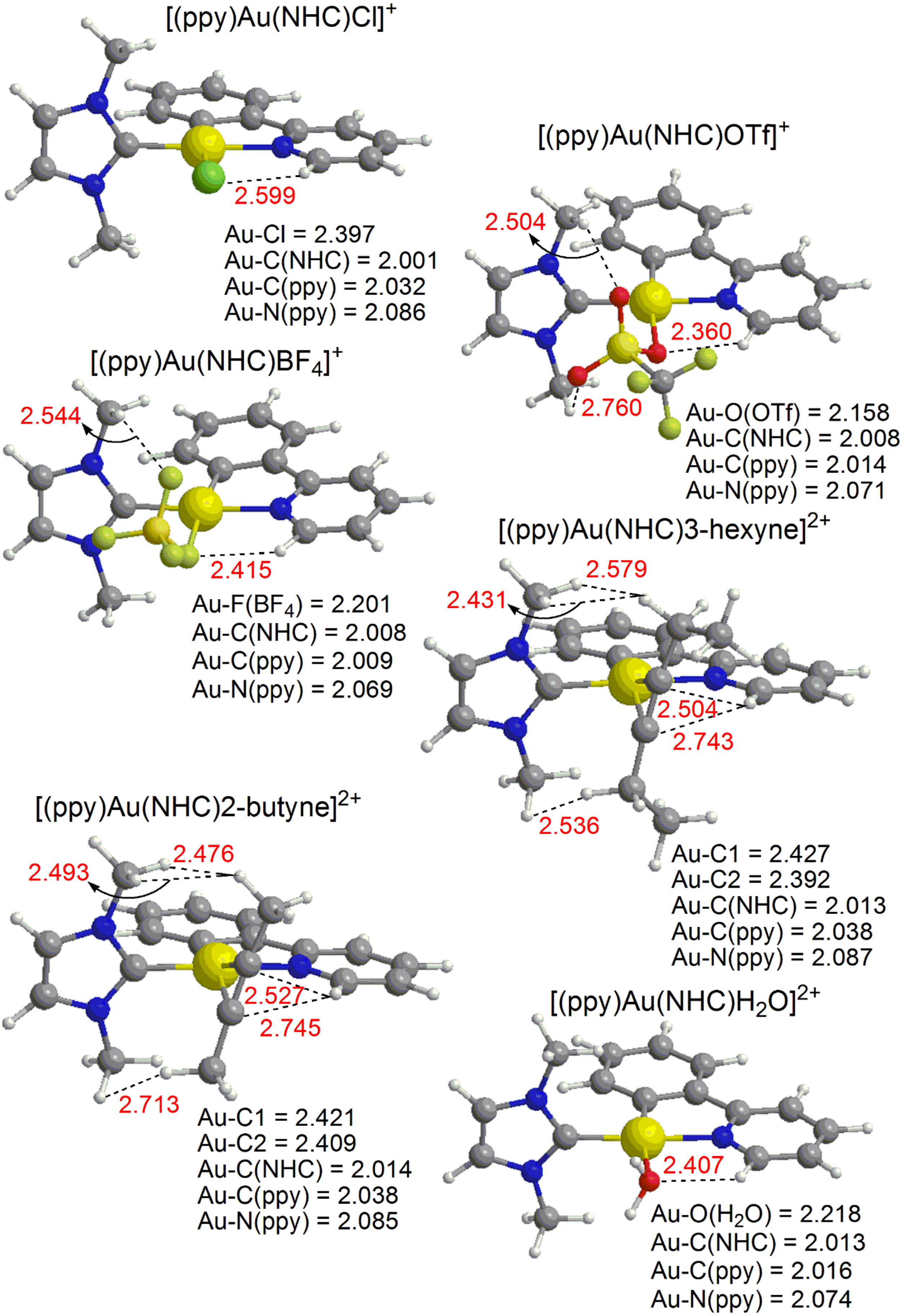
[(ppy)Au(NHC)Cl]$^+$
2.599
Au-Cl = 2.397
Au-C(NHC) = 2.001
Au-C(ppy) = 2.032
Au-N(ppy) = 2.086
[(ppy)Au(NHC)OTf]$^+$
2.504
2.360
2.760
Au-O(OTf) = 2.158
Au-C(NHC) = 2.008
Au-C(ppy) = 2.014
Au-N(ppy) = 2.071
[(ppy)Au(NHC)BF$_4$]$^+$
2.544
2.415
Au-F(BF$_4$) = 2.201
Au-C(NHC) = 2.008
Au-C(ppy) = 2.009
Au-N(ppy) = 2.069
[(ppy)Au(NHC)3-hexyne]$^{2+}$
2.579
2.431
2.504
2.743
2.536
Au-C1 = 2.427
Au-C2 = 2.392
Au-C(NHC) = 2.013
Au-C(ppy) = 2.038
Au-N(ppy) = 2.087
[(ppy)Au(NHC)2-butyne]$^{2+}$
2.493
2.476
2.527
2.745
2.713
Au-C1 = 2.421
Au-C2 = 2.409
Au-C(NHC) = 2.014
Au-C(ppy) = 2.038
Au-N(ppy) = 2.085
[(ppy)Au(NHC)H$_2$O]$^{2+}$
2.407
Au-O(H$_2$O) = 2.218
Au-C(NHC) = 2.013
Au-C(ppy) = 2.016
Au-N(ppy) = 2.074

Figure S14: Optimized structures of [(ppy)Au(NHC)X]$^{+/2+}$ (X = Cl^-, BF_4^-, OTf^-, H_2O, 2-butyne, 3-hexyne) complexes. Main bond lengths are reported (Å). Principal interaction contacts (below 3 Å) of the NHC and ppy moieties with X ligands are highlighted in red color.

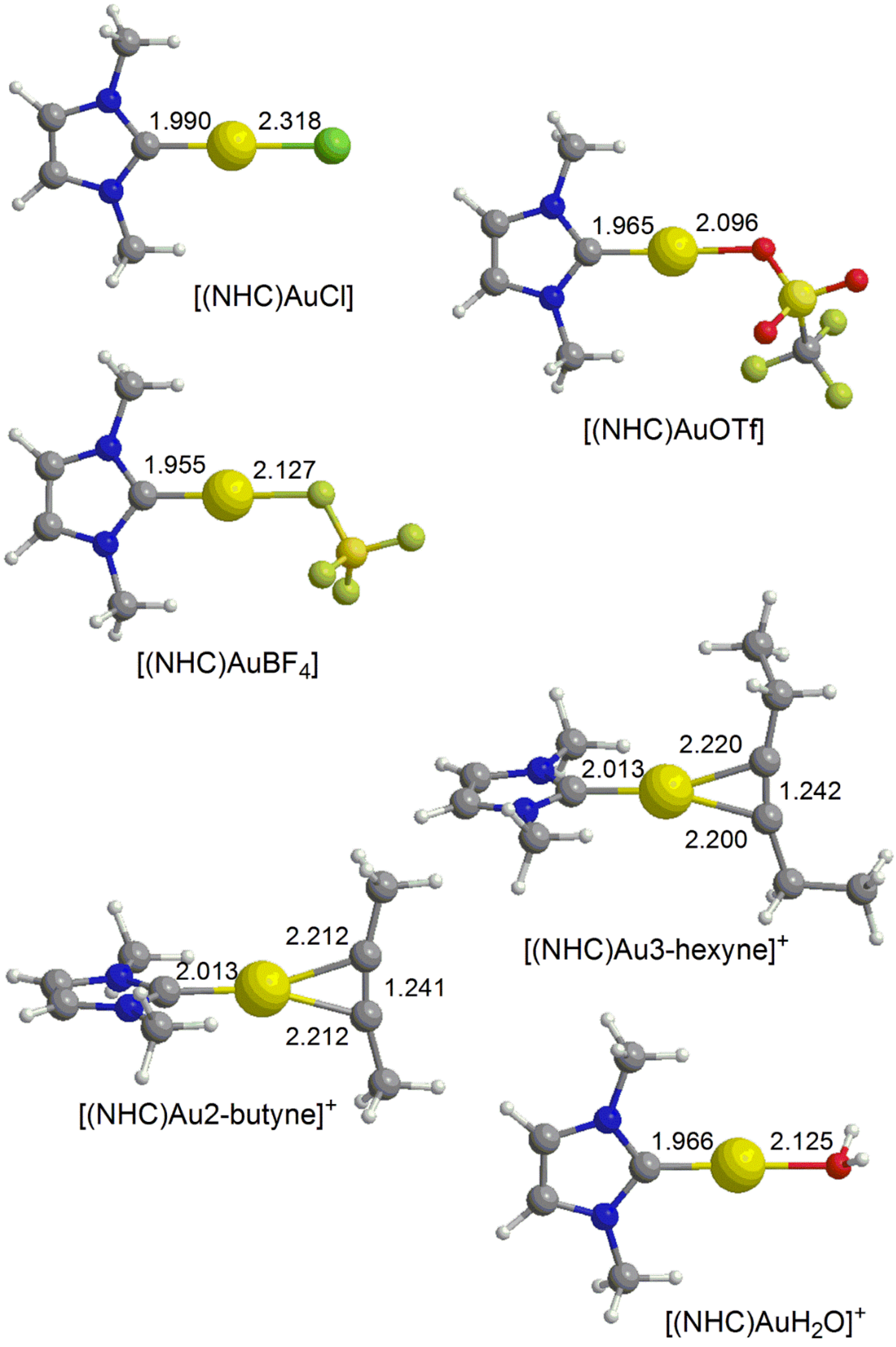

Figure S15 Optimized structures of $[(NHC)AuX]^{0/+}$ (X = Cl^-, BF_4^-, OTf^-, H_2O, 2-butyne, 3-hexyne) complexes. Main bond lengths are shown (Å).

The most relevant structural variation from the real to the model Au(III)Cl complex concerns the lengthening of the Au(III)-Cl bond distance from 2.380 (real) to 2.397 (model) Å which apparently seems to be not in line with the stronger stabilization of the model complex with respect to the real one (5.9 kcal/mol). The origin of the weaker interaction in the real Au(III)Cl complex can be mainly ascribed to the steric interaction between Cl and the two isopropylphenyl groups of IPr (see Figure S12). On comparing the coordination ability of BF_4^- and OTf^- to the metal center, a difference of only 5.0 kcal/mol is calculated in the Au(III) real complex, with OTf^- being more coordinating. Interestingly, in the Au(III) model complex such an energy difference increases to 10.0 kcal/mol. We can surmise that the higher steric hindrance in real complex due to the two isopropylphenyl groups of IPr is responsible for a larger destabilization of the Au(III)-OTf bond than the Au(III)-BF_4 one caused by the larger size and less "spherical" symmetry of OTf^- compared to BF_4^-. As a consequence, the coordination ability of OTf^- decreases in the Au(III) real complex, although it remains slightly more coordinating than BF_4^-.

However, the decreasing contribution of the steric hindrance to the OTf^- bonding energy could be counteracted by an additional effect due to the IPr and ppy ligands, namely the long-range noncovalent interactions. To understand this effect, we need to look closer at the geometries of the species shown in Figures S12 and S14, where the nearest contacts between the O and F atoms of the ligand X and the hydrogens from the ppy ring and the isopropylphenyl (methyl) groups of IPr (NHC) are highlighted. In general, fewer long-range interactions can be established between the coordinated anion and NHC than IPr, which are responsible for a different contribution to the overall bonding energy.

Considering the BF_4^- and OTf^- coordination in the Au(I) complex, the difference between their

bonding energies is −10.3 kcal/mol, similar to that between their bonding free energies in the Au(III) model complex (−10.0 kcal/mol). Thus, the Au(I) and Au(III) model systems seem to similarly interact with the two anions. This is a surprising result on the basis of the two charge unit difference between the two metal centres. Taking into account the linearity of the Au(I) complex and the absence of steric hindrance, which is also not greatly pronounced in the Au(III) model system, then probably the most important contribution to the OTf^- bonding energy in the Au(III) real system is the destabilizing steric effect of IPr.

Among the neutral ligands, H_2O exhibits the smallest calculated bonding energy in all the Au(III) real, Au(III) model and Au(I) complexes (Table S1 and Figure S13). On the basis of these results, water could be considered the weakest ligand in the series, in strong disagreement with the experiment. To rationalize these intriguing results we first compare the optimized structures of the $[(ppy)Au(IPr)X]^{2+}$ real and $[(ppy)Au(NHC)X]^{2+}$ (X = H_2O, 2-butyne, and 3-hexyne) model complexes depicted in Figures S12 and S14.

By comparing the Au(III)H_2O real and model geometries we can observe only slightly different bond distances. The most relevant structural variation from the Au(III) real to the model complex concerns the lengthening of H_2O oxygen-hydrogen from the ppy ring contact from 2.348 (real) to 2.407 (model) Å and the presence of long-range noncovalent interactions between H_2O hydrogens and the isopropylphenyl groups of IPr. The model complex is stabilized by 9.0 kcal/mol with respect to the real one, which can be ascribed to the removal of the steric interaction between water hydrogens and the two isopropylphenyl groups of IPr. Analogously, alkyne coordination shows large structural differences between the Au(III) real and Au(III) model complexes, mainly arising from the alkyne-IPr interactions. The larger bonding energy calculated for the alkynes with the model complex with respect to the real one could be then attributed to the decreasing of the NHC steric hindrance.

As a balance between the steric effects due to the IPr ligand and the stabilizing effects of long-range noncovalent interactions between alkyne and IPr and ppy, both the alkynes are found to be more

strongly coordinating to Au than water, with larger size 3-hexyne coordinating better than 2-butyne (see Table S1), thus indicating that the steric hindrance should not be the only effect that contributes to the selectivity of the metal towards the considered neutral ligands.

Compared to the Au(I) complexes geometries depicted in Figure S15, we generally observe shorter Au-OH_2, 2-butyne C≡C, and 3-hexyne C≡C bond distances (2.125, 2.212 and 2.220 Å, respectively) with respect to the corresponding Au(III) real and model complexes, associated with higher bonding energies (Table S1). This result indicates that steric effects are very important for water and alkyne coordination. In particular, the bonding energy difference between 2-butyne and 3-hexyne is only 1.9 kcal/mol, thus showing that the absence of steric hindrance/dispersion interactions makes the coordination ability of the two ligands almost identical.

Ligand effect on the coordination ability: alkynes vs water

[(ppy)AuCl3-hexyne]$^+$

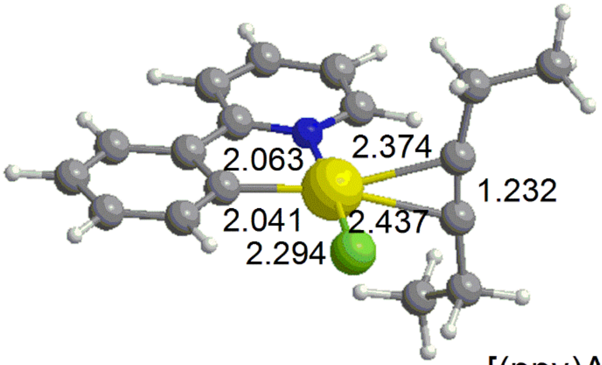

[(ppy)AuCl2-butyne]$^+$

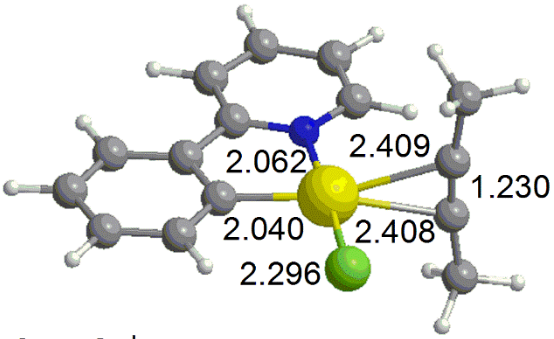

[(ppy)AuClH$_2$O]$^+$

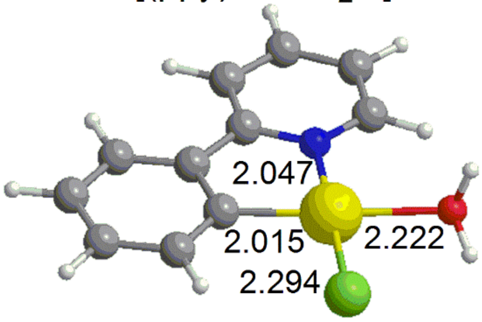

[(C^N^C)Au3-hexyne]$^+$

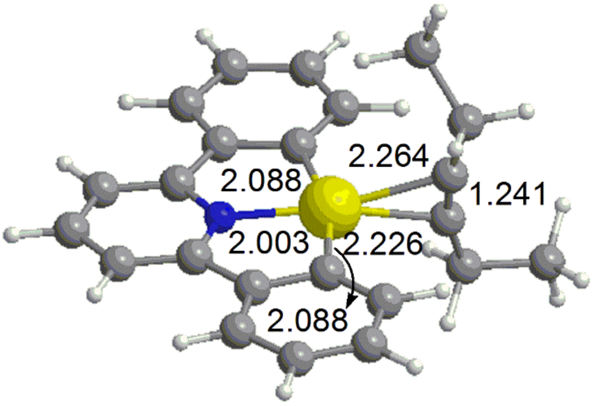

[(C^N^C)Au2-butyne]$^+$

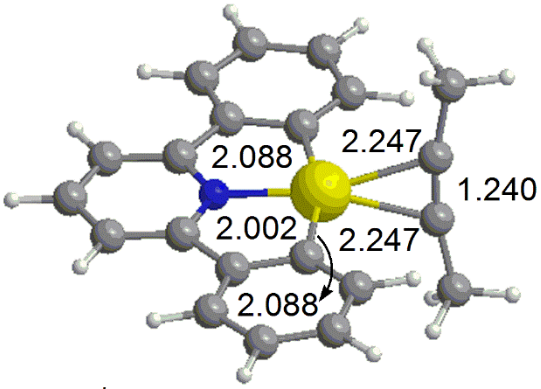

[(C^N^C)AuH$_2$O]$^+$

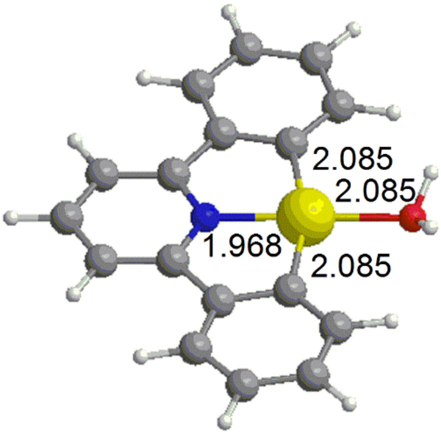

Figure 16. Optimized structures of [(C^N^C)AuX]+ and [(ppy)AuClX]+ (X = H_2O, 2-butyne, 3-hexyne) complexes. Bond distances are in Å.

In Table S2, X (X = H_2O, 3-hexyne, 2-butyne) bonding energies ΔE, enthalpies ΔH and Gibbs free energies ΔG (in kcal/mol) to $[(ppy)AuCl]^+$, $[(C\text{^}N\text{^}C)Au]^+$, $[(ppy)Au(IPr)]^{2+}$, $[(ppy)Au(NHC)]^{2+}$ and $[(NHC)Au]^+$ fragments are compared.

	$[(ppy)AuCl]^+$	$[(C\text{^}N\text{^}C)Au]^+$	$[(ppy)Au(IPr)]^{2+}$	$[(ppy)Au(NHC)]^{2+}$	$[(NHC)Au]^+$
	ΔE	ΔE	ΔE	ΔE	ΔE
H_2O	−19.6	−36.8	−15.2	−21.9	−28.4
3-hexyne	−29.4	−56.7	−31.4	−33.3	−43.6
2-butyne	−27.5	−54.1	−27.4	−31.1	−41.4
	ΔH	ΔH	ΔH	ΔH	ΔH
H_2O	−17.9	−34.9	−13.4	−18.7	−27.0
3-hexyne	−27.1	−54.2	−27.5	−30.4	−42.1
2-butyne	−25.3	−51.7	−23.8	−28.4	−40.2
	ΔG	ΔG	ΔG	ΔG	ΔG
H_2O	−6.7	−24.3	−0.4	−9.4	−16.4
3-hexyne	−17.3	−44.0	−15.9	−20.6	−31.3
2-butyne	−15.5	−40.9	−12.6	−19.6	−29.4

Table S2. X (X = H_2O, 3-hexyne, 2-butyne) bonding energies, enthalpies and Gibbs free energies (in kcal/mol) to $[(ppy)AuCl]^+$, $[(C\text{^}N\text{^}C)Au]^+$, $[(ppy)Au(IPr)]^{2+}$, $[(ppy)Au(NHC)]^{2+}$ and $[(NHC)Au]^+$.

Methodological study – explicit evaluation of dispersion and solvent effects on bonding energies and geometries of $[(ppy)Au(IPr)X]^{+/2+}$ and $[(ppy)Au(NHC)X]^{+/2+}$ (X = Cl^-, BF_4^-, OTf^-, H_2O, 2-butyne, 3-hexyne) complexes.

The methodological study where dispersion and solvation effects on bonding energies and geometries are explicitly evaluated is based on the B2PLYP-D//BP86 protocol, characterized by single point B2PLYP-D perturbatively corrected doubly hybrid functional calculations performed on the optimized BP86 gas phase structures (see Computational Details below) and analysis is given in terms of electronic bonding energies ΔE, which are shown to be good approximation to ΔH values and to qualitatively reproduce the ΔG values trend.

Computational Details

DFT calculations have been performed using the Amsterdam Density Functional (ADF) (2016 version)[4,5,6] and the related Quantum-regions Interconnected by Local Descriptions (QUILD)[7] program packages. For geometry optimization the GGA BP86 functional[8,9] (DFT/BP86) and a Slater-type TZ2P triple zeta basis set with two polarization functions for all atoms, in the small frozen core approximation, were used. Relativistic effects were included by the scalar zero-order regular approximation ZORA Hamiltonian[10,11,12] The Grimme 3 BJDAMP dispersion correction[13] was included in BP86 single point calculation (BP86-D3) in analyses aimed at explicitly evaluating the dispersion forces contribution to the energy.

Unless otherwise specified, all final energies have been computed by single point B2PLYP-D perturbatively corrected doubly hybrid functional[14] calculations performed on the optimized BP86 gas phase structures in conjunction with a def2-TZVP basis set for all atoms and an ECP pseudopotential for gold to include relativistic effects. This computational set up (referred as to B2PLYP-D//BP86) has been shown to be very accurate in describing catalysis by gold-containing

species in benchmark calculations.[15,16,17] with the B2PLYP-D functional, which includes also the dispersion terms of energy, using ORCA program.[18]

All calculations were carried out for the closed-shell singlet state. Solvation has been included by the Conductor like Screening Model COSMO,[19,20,21] using dichloromethane as the solvent, by single point BP86-D3 calculations on optimized BP86 gas phase structures.

Coordination ability – gas phase

We have performed calculations of electronic energy change (ΔE) at gas phase B2PLYP-D//BP86 level of theory for the following reactions:

$[(ppy)Au(IPr)]^{2+} + X^{-/0} \rightarrow [(ppy)Au(IPr)X]^{+/2+}$ (1)

$[(ppy)Au(NHC)]^{2+} + X^{-/0} \rightarrow [(ppy)Au(NHC)X]^{+/2+}$ (2)

$[(NHC)Au]^{+} + X^{-/0} \rightarrow [(NHC)AuX]^{0/+}$ (3)

The results are summarized in Table S3 and depicted in Figure S17, where $[(ppy)Au(IPr)]^{2+}$, $[(ppy)Au(NHC)]^{2+}$ and $[(NHC)Au]^{+}$ fragments are denoted for simplicity as "Au(III) real", "Au(III) model" and "Au(I)", respectively.

	ΔE Cl^-	ΔE OTf^-	ΔE BF_4^-	ΔE 3-hexyne	ΔE 2-butyne	ΔE H_2O
Au(III) real	−202.3	−158.5	−153.6	−30.0	−25.8	−21.5
Au(III) model	−225.0	−187.5	−176.5	−44.9	−39.2	−28.4
Au(I)	−157.0	−120.2	−110.1	−52.0	−50.2	−36.6

Table S3. X (X = Cl^-, OTf^-, BF_4^-, 3-hexyne, 2-butyne, and H_2O) bonding energies (ΔE in kcal/mol) to $[(ppy)Au(IPr)]^{2+}$ (Au(III) real), $[(ppy)Au(NHC)]^{2+}$ (Au(III) model) and $[(NHC)Au]^{+}$ (Au(I)).

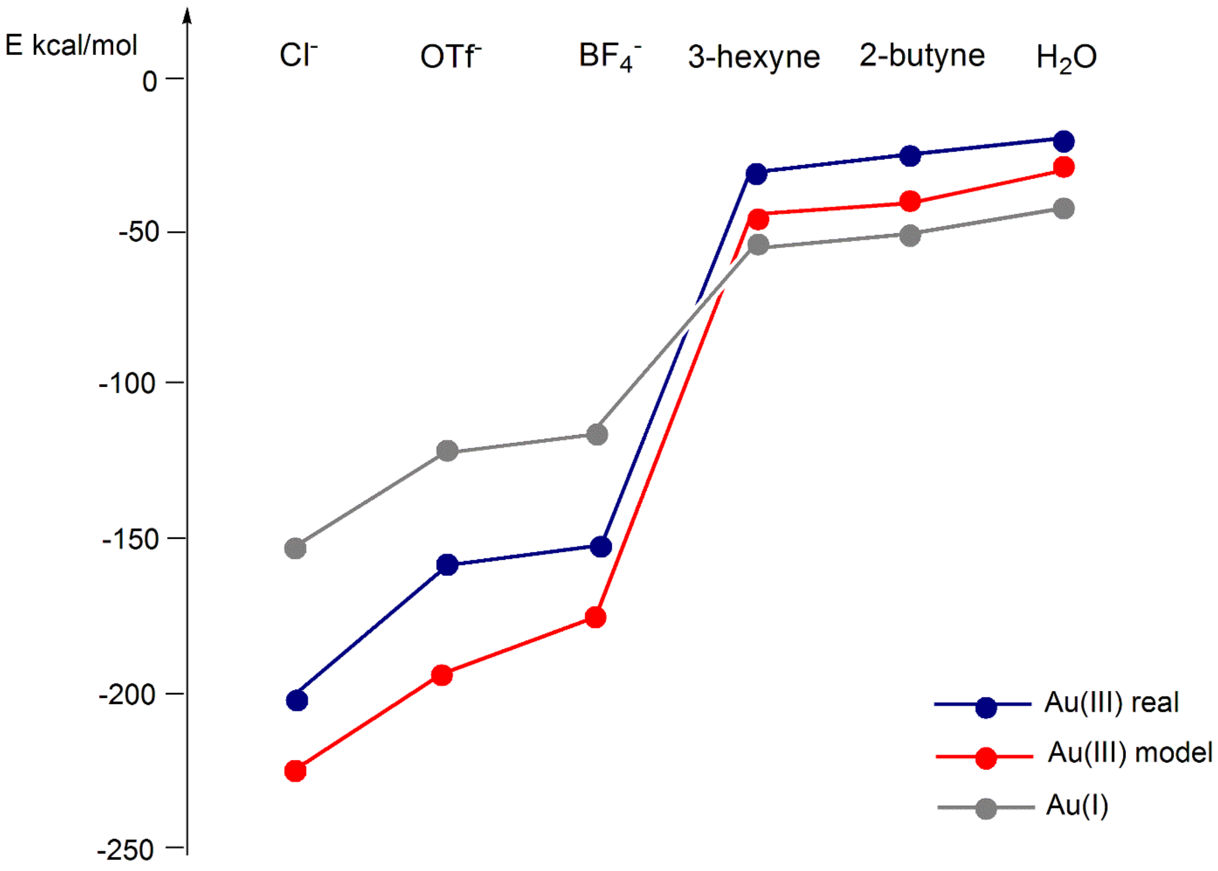

Figure S17. Bonding energy trend for real and model Au(III)X and for Au(I)X (X = Cl^-, OTf^-, BF_4^-, 3-hexyne, 2-butyne and H_2O) complexes. Plotted data are taken from Table S3.

Note that at the gas phase coordination ability trend is the same for Au(III) real, Au(III) model and Au(I) complexes, namely Cl^- > OTf^- > BF_4^- > 3-hexyne > 2-butyne > H_2O, for all the systems. Comparison of Table S3 and Figure S17 (B2PLYP-D//BP86) with Table S1 and Figure S13 (bottom) (BP86-D3 solv//BP86-D3 solv), shows that inclusion of solvent mainly affects the bonding energy trends within the anionic ligands subset, which are significantly reduced for all the Au(III) real, Au(III) model and Au(I) complexes. In particular, for the Au(III) real complexes, they decrease to such an extent as to be lower than those for the Au(I) complexes, thus suggesting a stronger impact for real Au(III)X (X = Cl^-, OTf^-, BF_4^-) complexes.

Dispersion and solvation effects on the bonding energies

In the following the dispersion and solvation effects will be evaluated on the bonding energies, using geometries optimized at the BP86 gas phase level.

Anionic ligands

The dispersion contribution for properly describing the noncovalent interactions, such as those playing a role in the anion-ligand interactions in the Au(III) real complexes, deserves a more detailed analysis. Actually, the bonding energies in Table S3 have been calculated using the B2PLYP-D functional which already includes dispersion. The dispersion effect on the bonding energy can be explicitly estimated by comparing the BP86 calculated bonding energies with those obtained by BP86 plus the Grimme dispersion correction (BP86-D3) for the Au(III) real and model systems. The results are reported in Table S4.

	ΔE (BP86) Au(III) real	ΔE (BP86-D3) Au(III) real	ΔE (BP86) Au(III) model	ΔE (BP86-D3) Au(III) Model	ΔE (BP86-D3) solv Au(III) real	ΔE (BP86-D3) solv Au(III) model
$[Au(III)Cl]^+$	−190.0	−196.8 (−202.3)	−214.0	−220.8 (−225.0)	−53.3	−60.2
$[Au(III)BF_4]^+$	−142.4	−150.7 (−153.6)	−167.0	−175.7 (−176.5)	−22.8	−32.6
$[Au(III)OTf]^+$	−140.2	−156.2 (−158.5)	−173.0	−185.8 (−187.5)	−33.0	−46.0
ΔE OTf^-/BF_4^-	2.2	−5.5 (−4.9)	−6.0	−10.1 (−11.0)	−10.2	−13.4

Table S4. X (X = Cl^-, BF_4^-, OTf^-) bonding energies (kcal/mol) to $[(ppy)Au(III)(IPr)]^{2+}$ (Au(III) real) and $[(ppy)Au(III)(NHC)]^{2+}$ (Au(III) model) complexes calculated at the BP86 (ΔE(BP86)), BP86 including the Grimme dispersion correction (ΔE(BP86-D3)) and BP86 including both the Grimme dispersion correction and solvent (ΔE(BP86-D3) solv) levels. Corresponding values calculated at the B2PLYP-D level are also shown in parentheses for comparison. Energy differences (ΔE OTf^-/BF_4^-) between coordination ability of OTf^- and BF_4^- for Au(III) real and Au(III) model at each level of theory are reported.

Remarkably, from BP86 calculations an energy difference between the BF_4^- and OTf^- Au(III) real systems of 2.2 kcal/mol is obtained with Au(III)BF_4 being more stable than Au(III)OTf. This result is completely different from that found at the B2PLYP-D level. Inclusion of Grimme D3 dispersion correction in the BP86 calculations gives an energy difference of −5.5 kcal/mol, with Au(III)OTf as the most stable complex. This result is close to that obtained with B2PLYP-D calculations (−4.9 kcal/mol) and shows the importance of the van der Waals dispersion forces in the anion coordination. These long-range interactions are thus demonstrated to play an important role in these large dimension systems where high polarization of the ligands fragments is expected.

The contribution of the dispersion forces to the bonding energy is generally lower for the Au(III) model systems, in agreement with the smaller number of noncovalent interactions arising when NHC replaces IPr. We can conclude that increasing the dimension of the ligands L and X the contribution of the dispersion forces to the total energy becomes increasingly relevant.

Solvation is also expected to have an impact on the bonding energies through modulation of dispersion interactions. In Table S4 values calculated at BP86-D3 level by including dichloromethane as solvent are shown. A general decrease of the bonding energies can be observed, although the trend of the gas phase BP86-D3 values (i.e. Au(III)Cl > Au(III)OTf > Au(III)BF_4) is retained for both Au(III) real and model complexes. Interestingly, the energy difference between Au(III)BF_4^- and Au(III)OTf^- real complexes increases to −10.2 kcal/mol, with Au(III)OTf^- as the most stable complex, as well as that between corresponding model complexes (−13.4 kcal/mol). Thus the dichloromethane effect appears to be beneficial for stabilizing the Au(III)OTf^- (both real and model) complex.

Neutral ligands

To further analyze the selectivity of the metal towards the considered neutral ligands, the effect of noncovalent interactions between H_2O, 2-butyne and 3-hexyne with IPr(NHC) and ppy ligands has been explicitly evaluated by comparing BP86 and BP86-D3 bonding energies. The results are shown in Table S5.

	ΔE (BP86) Au(III) real	ΔE (BP86-D3) Au(III) Real	ΔE (BP86) Au(III) model	ΔE (BP86-D3) Au(III) model	ΔE (BP86-D3) solv Au(III) Real	ΔE (BP86-D3) solv Au(III) model
$[Au(III)H_2O]^{2+}$	−14.8	−19.0 (−21.5)	−20.7	−25.5 (−28.4)	−12.5	−20.9
$[Au(III)3\text{-hexyne}]^{2+}$	−10.4	−34.4 (−30.0)	−30.0	−47.2 (−44.9)	−27.9	−32.8
ΔE H_2O/3-hexyne	4.4	−15.4 (−8.5)	−9.3	−21.7 (−16.5)	−15.4	−11.9
$[Au(III)2\text{-butyne}]^{2+}$	−9.6	−29.3 (−25.8)	−28.0	−41.9 (−39.2)	−25.1	−31.3
ΔE H_2O/2-butyne	5.2	−10.3 (−4.3)	−7.3	−16.4 (−10.8)	−12.6	−10.4

Table S5. X (X = H_2O, 3-hexyne, 2-butyne) bonding energies (kcal/mol) to $[(ppy)Au(IPr)]^{2+}$ (Au(III) real) and $[(ppy)Au(NHC)]^{2+}$ (Au(III) model) complexes calculated at the BP86 (ΔE(BP86)), BP86 including the Grimme dispersion correction (ΔE(BP86-D3)) and BP86 including both the Grimme dispersion correction and solvent (ΔE(BP86-D3 solv)) levels. Corresponding values calculated at the B2PLYP-D level are also shown in parentheses for comparison. Energy differences between coordination ability of H_2O and 3-hexyne (ΔE H_2O/3-hexyne) and of H_2O and 2-butyne (ΔE H_2O/2-butyne) for Au(III) real and Au(III) model at each level of theory are reported.

Interestingly, for the Au(III) real complex the BP86 bonding energy is lower for both 2-butyne and 3-hexyne than for H_2O, with a bonding energy difference of 5.2 and 4.4 kcal/mol, respectively. Inclusion of Grimme dispersion correction changes the result: the BP86-D3 bonding energy is higher for both 2-butyne and 3-hexyne than for H_2O, with a bonding energy difference of −10.3 and −15.4 kcal/mol, respectively (−4.3 and −8.5 kcal/mol at B2PLYP-D level, respectively). For the Au(III) alkyne model complexes, the dispersion contribution to the bonding energy is generally lower than that

calculated for the corresponding Au(III) real complexes, whereas is nearly negligible for Au(III)H_2O model (compare bonding energies at BP86 and BP86-D3 values in Table S5). These results show again that the dispersion contribution is more relevant for the larger size ligand (IPr vs. NHC). Overall, this analysis highlights the crucial contribution of the dispersion forces to the total bonding energy also for neutral ligands. In addition, these results also demonstrate that dispersion forces are responsible for the bonding energy difference between the two alkynes, making the 3-hexyne more coordinating than 2-butyne. Inclusion of solvent effect generally lowers the bonding energies for both Au(III) real and Au(III) model complexes, although the energy differences between H_2O and alkynes are only slightly affected for Au(III) real complexes (compare BP86-D3 and BP86-D3 solv ΔE H_2O/3-hexyne and ΔE H_2O/2-butyne values in Table S5). The same general trend of the gas phase BP86-D3 values (i.e. Au(III)3-hexyne > Au(III)2-butyne > Au(III)H_2O) is found for both Au(III) real and model complexes. It is worth noting that dichloromethane further lowers the bonding energy of Au(III)H_2O real and model complexes from −19.0 to −12.5 kcal/mol and from −25.5 to −20.9 kcal/mol, respectively. Overall, from Tables S4 and S5, inclusion of both dispersion and solvent (BP86-D3 solv) on the gas phase optimized BP86 geometries leads to the following ΔE trends: Cl^- > OTf^- > 3-hexyne > 2-butyne > BF_4^- > H_2O for Au(III) real, and Cl^- > OTf^- > 3-hexyne ≈ BF_4^- ≈ 2-butyne > H_2O for Au(III) model.

Dispersion and solvation effects on the geometries

Dispersion and solvation effects on the geometries can be readily evaluated by comparing ΔE values for Au(III) real and model complexes optimized at the BP86-D3 solv level (Table S1) with those for the same complexes optimized at the BP86 level with energies calculated by including both dispersion and solvation (BP86-D3 solv) (Tables S4 and S5). This is visualized in Figure S18.

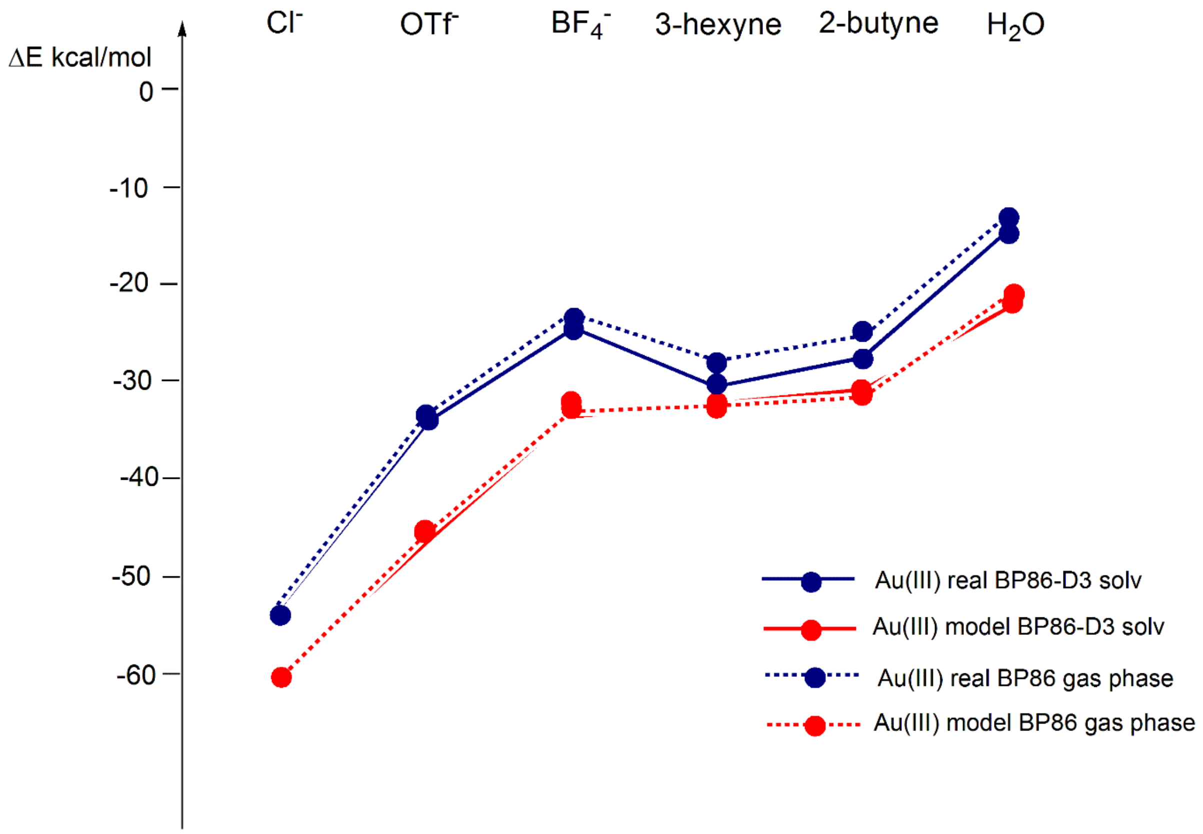

Figure S18. Bonding energy trend for real and model Au(III)X (X = Cl^-, OTf^-, BF_4^-, 3-hexyne, 2-butyne and H_2O) complexes optimized at BP86-D3 solv level (solid lines) and at BP86 gas phase with energies calculated by including both dispersion and solvent (BP86-D3 solv) level (dashed lines). Plotted data are taken from Tables S1, S4 and S5.

From Figure S18 we can immediately see that the gas-phase optimized structures can be considered as a good approximation for Au(III) model complexes and still reasonable for Au(III) real complexes. Additional detailed benchmark calculations for dispersion and solvation effects on geometries have been performed. Results are collected in Figure S19 and Table S6. In Figure S19 optimized structures of the $[(ppy)Au(IPr)X]^{+/2+}$ real and $[(ppy)Au(NHC)X]^{+/2+}$ model complexes (X = Cl^-, H_2O, 2-butyne) obtained by dispersion (BP86-D3) and solvent (BP86-D3 solv) inclusion are compared. Relative energies with respect to the corresponding optimized geometries calculated at the gas-phase

(BP86/BP86-D3 and BP86/BP86-D3 solv) are also reported. Inclusion of both dispersion and solvation stabilizes the Au(III) model complexes structures by −0.4/−1.6 kcal/mol, with a larger effect on the Au(III) real complexes structures, which are stabilized by −4.3/−6.0 kcal/mol.

In Table S6 bonding energies calculated with BP86 plus the Grimme dispersion correction optimized geometries (BP86-D3) and with BP86 plus the Grimme dispersion correction and solvent optimized geometries (BP86-D3 solv) of reactants and products are compared to those calculated with BP86 optimized geometries (values taken from Tables S4 and S5). Results show that both dispersion and solvation only slightly affect the bonding energies. Remarkably, the same coordination trend obtained with the gas phase structures (i.e. Au(III)Cl > Au(III)2-butyne > Au(III)H_2O) is quantitatively found for both Au(III) real and model complexes. Therefore, gas-phase optimized structures can be considered as a good approximation for the analysed complexes.

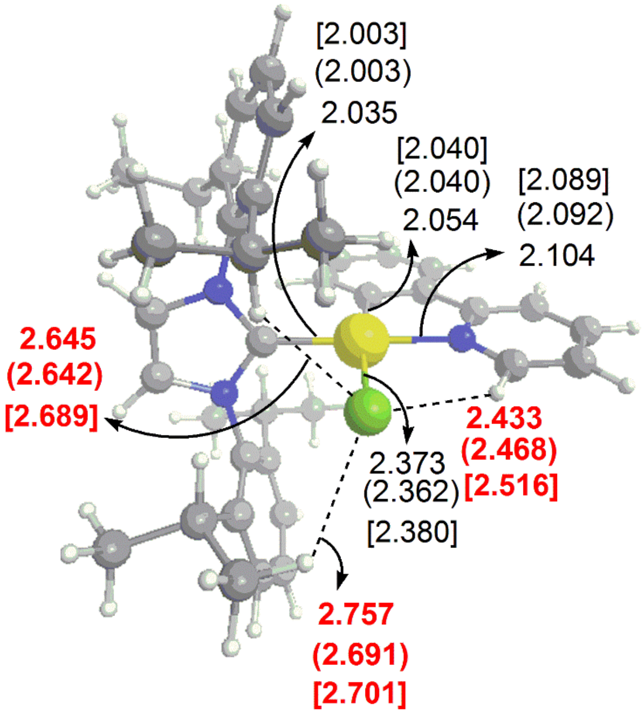

(-3.4 kcal/mol) [-4.3 kcal/mol]

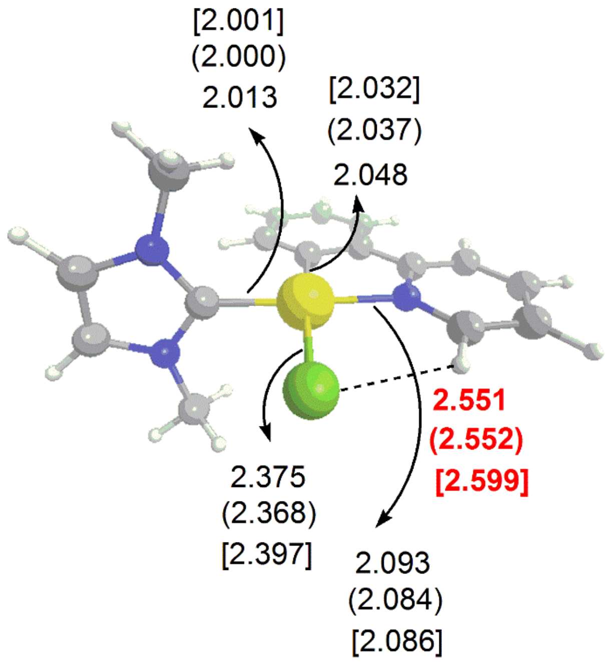

(-0.2 kcal/mol) [-0.4 kcal/mol]

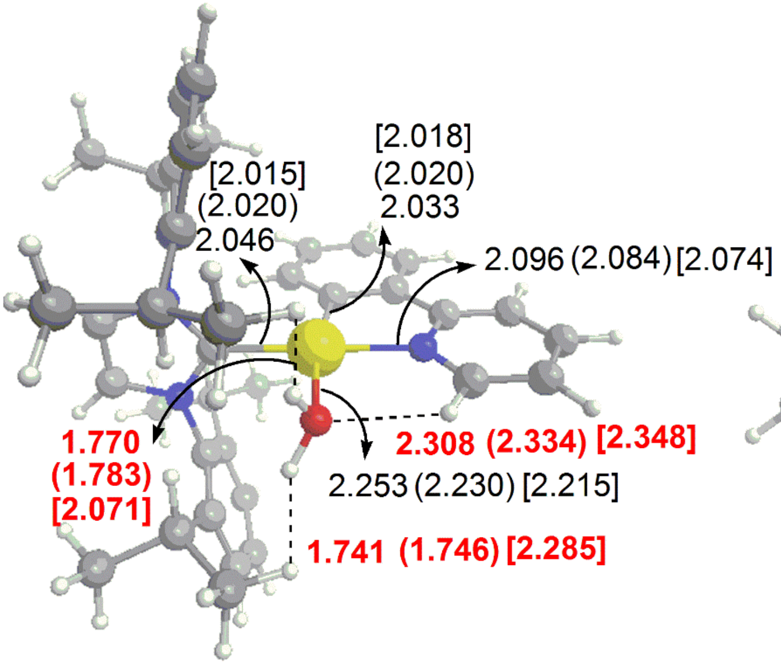

(-3.1 kcal/mol) [-6.0 kcal/mol]

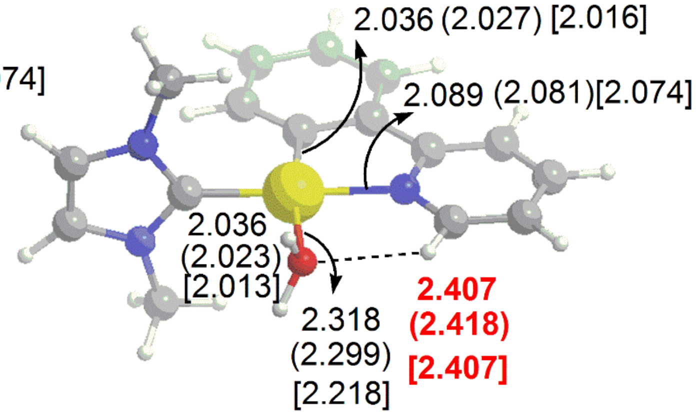

(-0.3 kcal/mol) [-1.6 kcal/mol]

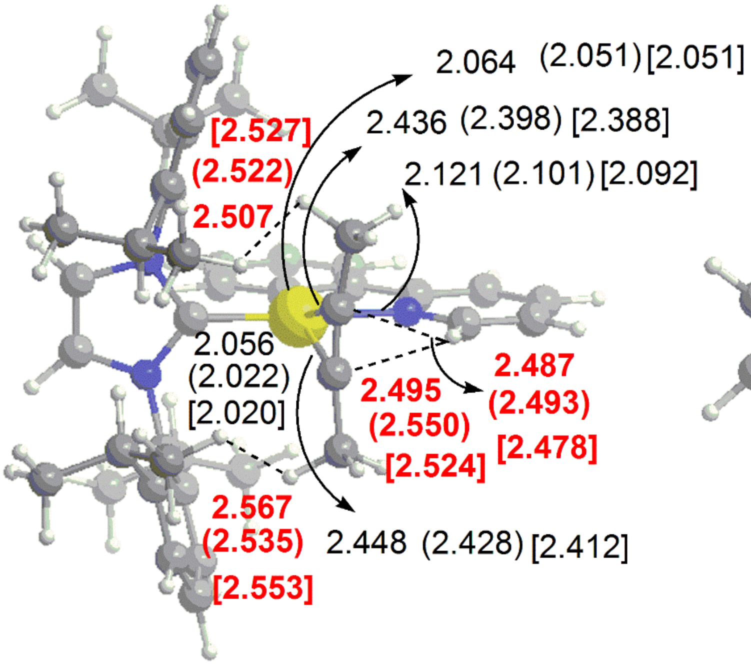

(-4.5 kcal/mol) [-5.3 kcal/mol]

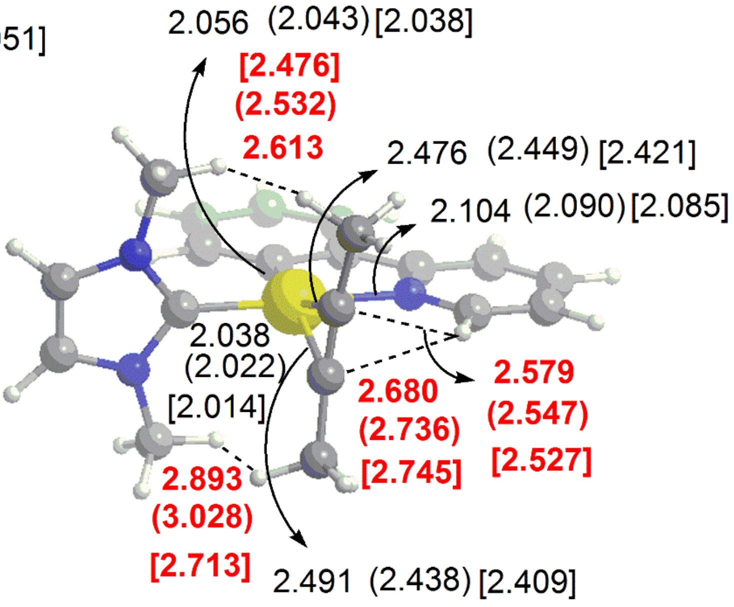

(-0.5 kcal/mol) [-1.1 kcal/mol]

Figure S19: Optimized structures of: top) Au(III)Cl real and Au(III)Cl model complexes; middle) Au(III)H_2O real and Au(III)H_2O model complexes; and bottom) Au(III)2-butyne real and Au(III)2-butyne model complexes. Distances are in Å. Main interaction contacts of the IPr (NHC) and ppy moieties with X (X = Cl^-, H_2O, 2-butyne) ligands are highlighted in red color. Values in round brackets refer to optimization calculation results including Grimme dispersion correction (BP86-D3), values in square brackets refer to optimization calculation results including both Grimme dispersion correction and solvent effect (COSMO) (BP86-D3 solv). Values referring to gas phase BP86 optimized geometries are also reported without brackets for comparison.

	ΔE (BP86-D3) Au(III) real	ΔE (BP86-D3) Au(III) model	ΔE (BP86-D3) solv Au(III) real	ΔE (BP86-D3) solv Au(III) model
$[Au(III)Cl]^+$	−197.7 (−196.8)	−220.7 (−220.8)	−54.3 (−53.3)	−59.3 (−60.2)
$[Au(III)H_2O]^{2+}$	−19.6 (−19.0)	−25.5 (−25.5)	−15.2 (−12.5)	−21.9 (−20.9)
$[Au(III)2\text{-butyne}]^{2+}$	−31.3 (−29.3)	−42.2 (−41.9)	−27.4 (−25.1)	−31.1 (−31.3)

Table S6. X (X = Cl^-, H_2O, 2-butyne) bonding energies (kcal/mol) to $[(ppy)Au(IPr)]^{2+}$ (Au(III) real) and $[(ppy)Au(NHC)]^{2+}$ (Au(III) model) complexes calculated at the BP86 including the Grimme dispersion correction (ΔE(BP86-D3)) and BP86 including both the Grimme dispersion correction and solvent (ΔE(BP86-D3 solv)) optimized geometries. Corresponding values calculated using the gas phase BP86 optimized geometries are also shown in parentheses for comparison (values taken from Table S4 and Table S5).

Validation of ΔE bonding analysis

	ΔE (BP86-D3) solv Au(III) model	ΔH (BP86-D3) solv Au(III) model	ΔG (BP86-D3) solv Au(III) model
$[Au(III)Cl]^{+}$	−60.2	−58.1	−59.2
$[Au(III)BF_4]^{+}$	−32.6	−30.9	−19.2
$[Au(III)OTf]^{+}$	−46.0	−44.2	−30.1
**Δ OTf^-/BF_4^-**	**−13.4**	**−13.3**	**−10.9**
$[Au(III)H_2O]^{2+}$	−20.9	−19.3	−8.5
$[Au(III)3\text{-hexyne}]^{2+}$	−32.8	−31.0	−17.6
**Δ H_2O/3-hexyne**	**−11.9**	**−11.7**	**−9.1**
$[Au(III)2\text{-butyne}]^{2+}$	−31.3	−29.6	−18.5
**Δ H_2O/2-butyne**	**−10.4**	**−10.3**	**−10.0**

Table S7. X (X = Cl^-, BF_4^-, OTf^-, H_2O, 3-hexyne, 2-butyne) bonding electronic energies ΔE, bonding enthalpies ΔH and bonding Gibbs free energies ΔG (kcal/mol) to $[(ppy)Au(III)(NHC)]^{2+}$ (Au(III) model) complexes calculated at the BP86 including both the Grimme dispersion correction and solvent (ΔE (BP86-D3 solv)) level on BP86 optimized geometries. Corresponding energy differences between coordination ability of OTf^- and BF_4^- (Δ OTf^-/BF_4^-), H_2O and 3-hexyne (Δ H_2O/3-hexyne) and H_2O and 2-butyne (Δ H_2O/2-butyne) are also reported.

In Table S7 bonding electronic energies ΔE, bonding enthalpies ΔH and bonding Gibbs free energies ΔG (kcal/mol) of all the considered ligands X (X = Cl^-, BF_4^-, OTf^-, H_2O, 3-hexyne, 2-butyne) to the $[(ppy)Au(III)(NHC)]^{2+}$ (Au(III) model) complex calculated at the BP86 level including both Grimme dispersion correction and solvent (BP86-D3 solv) on BP86 optimized geometries are compared.

Results show that electronic bonding energies are a good approximation to the bonding enthalpy values and a reasonable approximation for the bonding free energies. Notably, energy differences between coordination ability of OTf^- and BF_4^- (Δ OTf^-/BF_4^-), H_2O and 3-hexyne (Δ H_2O/3-hexyne) and H_2O and 2-butyne (Δ H_2O/2-butyne) are very close in terms of ΔE, ΔH and ΔG.

Coordination ability – ligand effect

Analogously to $[(ppy)Au(IPr)]^{2+}$, the electronic bonding energies of X (X = H_2O, 2-butyne, 3-hexyne) have been calculated at gas phase B2PLYP-D//BP86 level of theory for the following reactions:

$[(C^\wedge N^\wedge C)Au]^+ + X \rightarrow [(C^\wedge N^\wedge C)AuX]^+$ (4)

$[(ppy)AuCl]^+ + X \rightarrow [(ppy)AuClX]^+$ (5)

	ΔE<br>$[(ppy)AuCl]^+$	ΔE<br>$[(C^\wedge N^\wedge C)Au]^+$	ΔE<br>Au(III) real	ΔE<br>Au(III) model	ΔE<br>Au(I)
H_2O	−26.6	−42.9	−21.5	−28.4	−36.6
3-hexyne	−36.3	−64.7	−30.0	−44.9	−52.0
2-butyne	−33.5	−61.1	−25.8	−39.2	−50.2

Table S8. X (X = H_2O, 3-hexyne, 2-butyne) bonding energies (ΔE in kcal/mol) to $[(ppy)AuCl]^+$, $[(C^\wedge N^\wedge C)Au]^+$, $[(ppy)Au(IPr)]^{2+}$ (Au(III) real), $[(ppy)Au(NHC)]^{2+}$ (Au(III) model) and $[(NHC)Au]^+$ (Au(I)) in all the complexes.

The overall energetic trend in all the systems can be readily visualized in Figure S20, which qualitatively nicely compares with Figure 1 in the main text.

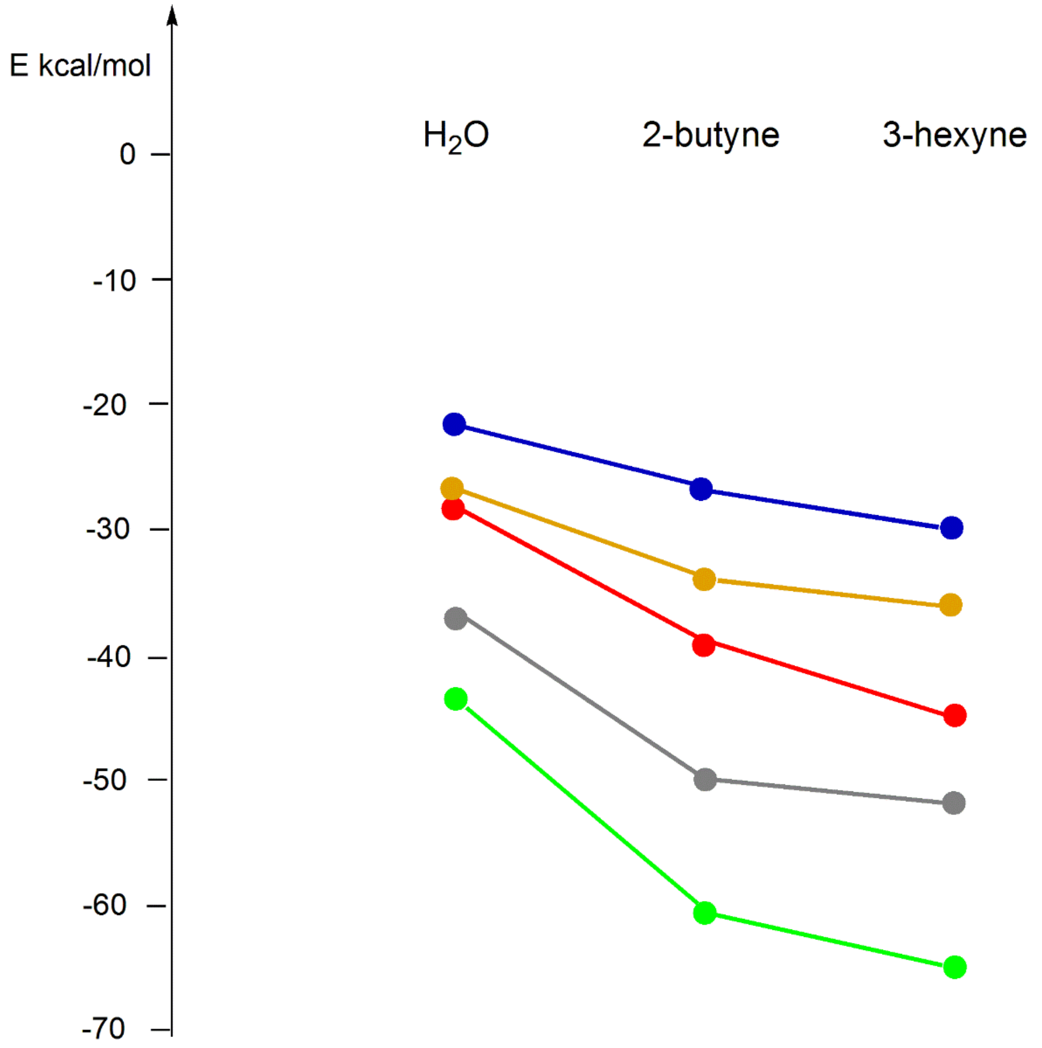

Figure S20: Bonding energy trend for [(ppy)AuClX]$^+$ (orange line), [(C^N^C)AuX]$^+$ (green line) [Au(III)X]$^{2+}$ real (blue line), [Au(III)X]$^{2+}$ model (red line) and [Au(I)X]$^+$ (grey line) complexes (X = H_2O, 3-hexyne, 2-butyne). Plotted data are taken from Table S1 and Table S8.

Bonding energies of $[(ppy)AuCl]^+$, $[(C^\wedge N^\wedge C)Au]^+$, Au(III) real, Au(III) model and Au(I) with X = H_2O, 3-hexyne and 2-butyne have been calculated at BP86-D3 solv level of theory to analyze the solvent effect. Results are collected in Table S9.

	ΔE (BP86-D3) solv $[(ppy)AuCl]^+$	ΔE (BP86-D3) solv $[(C^\wedge N^\wedge C)Au]^+$	ΔE (BP86-D3) solv Au(III) real	ΔE (BP86-D3) solv Au(III) model	ΔE (BP86-D3) solv Au(I)
H_2O	−19.2	−36.3	−12.5	−20.9	−28.2
3-hexyne	−29.0	−56.8	−27.9	−32.8	−42.5
2-butyne	−27.5	−53.9	−25.1	−31.3	−41.5

Table S9. X (X = H_2O, 3-hexyne, 2-butyne) bonding energies (ΔE in kcal/mol) to $[(ppy)AuCl]^+$, $[(C^\wedge N^\wedge C)Au]^+$, $[(ppy)Au(IPr)]^{2+}$ (Au(III) real), $[(ppy)Au(NHC)]^{2+}$ (Au(III) model) and $[(NHC)Au]^+$ (Au(I)) calculated at the BP86-D3 solv level of theory in all the complexes.

As it can be observed, inclusion of solvent generally lowers the bonding energies, without changing the qualitative trend. Note that even inclusion of dichloromethane suggests that Au(III) is not more oxophilic than Au(I), i.e. bonding energies with H_2O are always lower than those with alkynes for all the considered ligands.

Substitution of H_2O with the substrate at gas-phase B2PLYP-D//BP86 level: the pre-equilibrium step

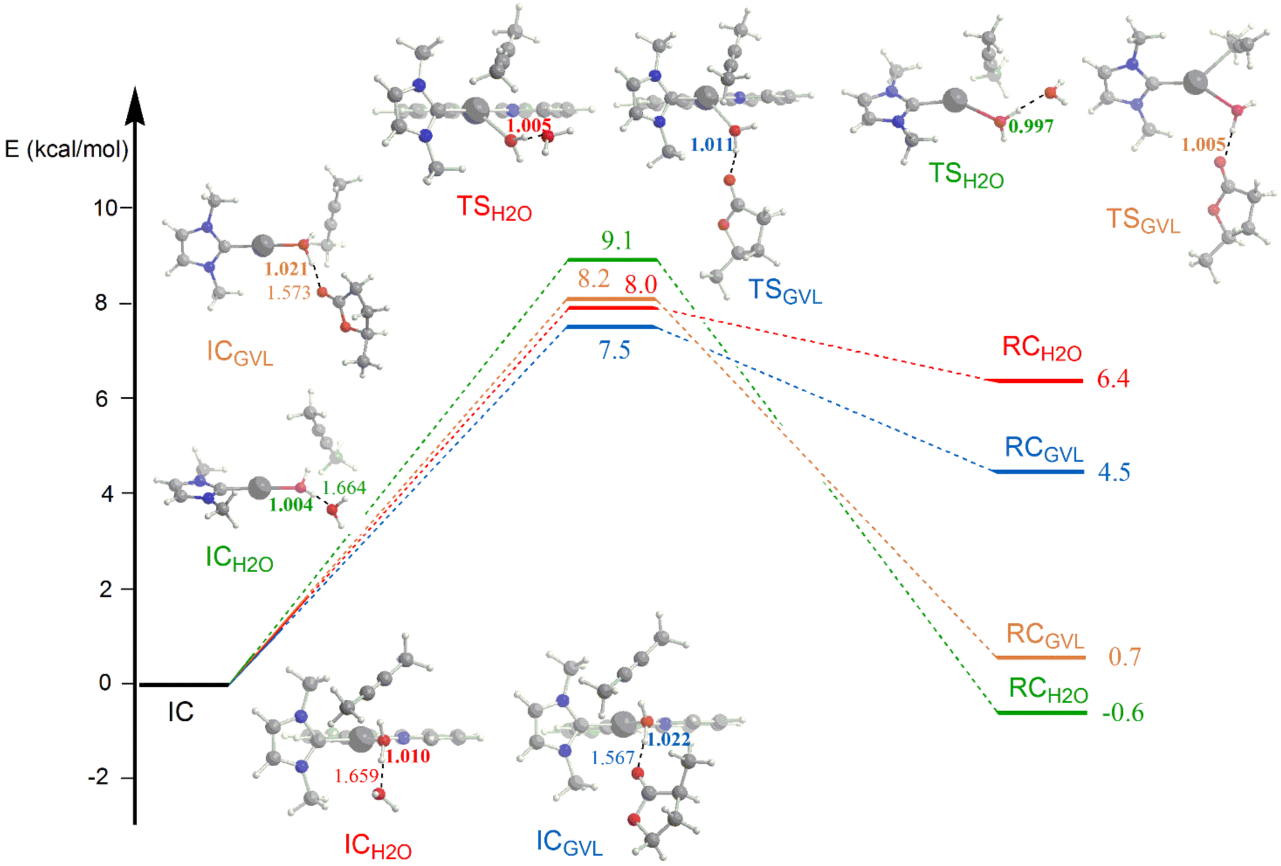

Figure S21: Energy profile for the pre-equilibrium step of the 2-butyne hydration reaction catalyzed by $[(ppy)Au(NHC)]^{2+}$ and [NHCAu]+ complexes in water (Au(III) red line, Au(I) green line) and in GVL (Au(III) blue line, Au(I) orange line) as solvent. Optimized structures of IC, TS and RC with relevant distances (in Å) are shown. IC has been taken as zero reference energy for all profiles.

	$\Delta E^{\neq}$ (BP86)	$\Delta G^{\neq}$ (BP86)
$TS_{H2O} - IC_{H2O}$ Au(III) model	14.2 (8.0)	15.7
TS_{GVL}-IC_{GVL} Au(III) model	12.0 (7.5)	12.3
$TS_{H2O} - IC_{H2O}$ Au(I)	13.0 (9.1)	12.5
$TS_{GVL} - IC_{GVL}$ Au(I)	11.6 (8.2)	14.0

Table S10. Activation electronic energies $\Delta E^{\neq}$ and activation Gibbs free energies $\Delta G^{\neq}$ (kcal/mol) values for the pre-equilibrium step of the 2-butyne hydration reaction catalysed by $[(ppy)Au(III)(NHC)]^{2+}$ (Au(III) model) and $[NHCAu]^{+}$ complexes in water ($TS_{H2O} - IC_{H2O}$) and in GVL (TS_{GVL}–IC_{GVL}) as solvent calculated at the BP86 level. Corresponding activation electronic energies $\Delta E^{\neq}$ values calculated at the B2PLYP-D//BP86 level are also reported in parenthesis for comparison (see Figure S21).

From Table S10 one can see that the activation electronic energies $\Delta E^{\neq}$ are good approximations for the activation Gibbs free energies $\Delta G^{\neq}$ values. Instead, inclusion of dispersion does affect the activation electronic energy barriers, by lowering them significantly.

In conclusion, this methodological study based on gas phase optimized geometries (BP86) shows that inclusion of dispersion is fundamental for calculating reliable bonding energy trends for the considered complexes and that solution phase results give qualitatively the same trends as those obtained in the gas phase, although the absolute values of the bonding energies are different.

However, for a more quantitative and accurate description of the bonding energy trends, both dispersion and solvation effects should be included in the optimization procedure, since they can affect the results, particularly for Au(III) real complexes. For this reason, the BP86 + D3 solv protocol for geometry optimizations has been used in the manuscript.

References

[1] Hintermann, L. Expedient Syntheses of the N-Heterocyclic Carbene Precursor Imidazolium Salts IPr.HCl, IMes.HCl and IXy.HCl. *Beilstein J. Org. Chem.* **2007**, *3* (22), 2-6.

[2] Janzen, D.E.; Doherty, S.R.; Vanderveer, D.G.; Hinkle, L.M.; Benefield, D.A.; Vashi, H.M.; Grant, G.J. Cyclometallated Gold(III) Complexes with a Trithiacrown Ligand: Solventless Au(III) Cyclometallation, Intramolecular Gold-Sulfur Interactions, and Fluxional Behavior in 1,4,7 – Trithiacyclononane Au(III) Complexes. *J. Organomet. Chem.* **2014**, *755*, 47-57.

[3] a) Ciancaleoni, G.; Belpassi, L.; Zuccaccia, D.; Tarantelli, F.; Belanzoni, P. Counterion effect in the reaction mechanism of NHC gold(I)-catalyzed alkoxylation of alkynes: computational insight into experiment. *ACS Catal.* **2015**, *5*, 803-814. b) Gatto, M.; Belanzoni, P.; Belpassi, L.; Biasiolo, L.; Del Zotto, A.; Tarantelli, F.; Zuccaccia, D. Solvent-, silver-, and acid-free NHC-Au-X catalyzed hydration of alkynes. The pivotal role of the counterion. *ACS Catal.* **2016**, *6*, 7363-7376. c) D'Amore, L.; Ciancaleoni, G.; Belpassi, L.; Tarantelli, F.; Zuccaccia, D.; Belanzoni, P. Unraveling the anion/ligand interplay in the reaction mechanism of gold(I)-catalyzed alkoxylation of alkynes. *Organometallics* **2017**, *36*, 2364–2376. d) Gatto, M.; Baratta, W.; Belanzoni, P.; Belpassi, L.; Del Zotto, A.; Tarantelli, F.; Zuccaccia, D. Hydration and alkoxylation of alkynes catalyzed by NHC-Au-OTf. *Green Chem.* **2018**, *20*, 2125-2134. e) Biasiolo, L.; Trinchillo, M.; Belanzoni, P.; Belpassi, L.; Busico, V.; Ciancaleoni, G.; D'Amora, A.; Macchioni, A.; Tarantelli, F.; Zuccaccia, D. Unexpected anion effect in the alkoxylation of alkynes catalyzed by N-Heterocyclic Carbene (NHC) cationic gold complexes. *Chem.-Eur. J.* **2014**, *20*, 14594-14598.

[4] SCM, Theoretical Chemistry, *ADF User's Guide. Release 2016*, Vrije Universiteit, Amsterdam, The Netherlands, 2016. http://www.scm.com.

[5] Fonseca Guerra, C.; Snijders, J.G.; te Velde, G.; Baerends, E.J. Towards an order-N DFT method. *Theor. Chem. Acc*. **1998**, *99*, 391-403.

[6] te Velde, G.; Bickelhaupt, F.M.; Baerends, E.J.; Fonseca Guerra, C.; van Gisbergen, S.J.A.; Snijders, J.G.; Ziegler, T. Chemistry with ADF. *J. Comput. Chem*. **2001**, *22*, 931-967.

[7] Swart, M.; Bickelhaupt, F.M. QUILD: quantum-regions interconnected by local descriptions. *J. Comput. Chem.* **2008**, *29*, 724-734.

[8] Becke, A.D. Density-functional exchange-energy approximation with correct asymptotic behavior. *Phys. Rev. A* **1988**, *38*, 3098-3100.

[9] Perdew, J.P. Density-functional approximation for the correlation energy of the inhomogeneous electron gas. *Phys. Rev. B* **1986**, *33*, 8822-8824.

[10] van Lenthe, E.; Baerends, E.J.; Snijders, J.G. Relativistic regular two-component Hamiltonians. *J. Chem. Phys*. **1993,** *99*, 4597-4610.

[11] van Lenthe, E.; Baerends, E.J.; Snijders, J.G. Relativistic total energy using regular approximations. *J. Chem. Phys*. **1994,** *101*, 9783-9792.

[12] van Lenthe, E.; Ehlers, A.; Baerends, E.J. Geometry optimizations in the zero order regular approximation for relativistic effects. *J. Chem. Phys*. **1999,** *110*, 8943-8953.

[13] Grimme, S.; Ehrlich, S.; Goerigk, L. Effect of the damping function in dispersion corrected density functional theory. *J. Comput. Chem.* **2011**, *32*, 1456-1465.

[14] Grimme, S. Semiempirical hybrid density functional with perturbative second-order correlation. *J. Chem. Phys.* **2006**, *124*, 034108.

[15] Ciancaleoni, G.; Rampino, S.; Zuccaccia, D.; Tarantelli, F.; Belanzoni, P.; Belpassi, L. An ab initio benchmark and DFT validation study on gold(I)-catalyzed hydroamination of alkynes. *J. Chem. Theory Comput.* **2014**, *10*, 1021-1034.

[16] Kang, R.; Chen, H.; Shaik, S.; Yao, J. Assessment of theoretical methods for complexes of gold(I) and gold(III) with unsaturated aliphatic hydrocarbon: which density functional should we choose? *J. Chem. Theory Comput.* **2011**, *7*, 4002-4011.

[17] Kang, R.; Lai, W.; Yao, J.; Shaik, S.; Chen, H. How accurate can a local coupled cluster approach be in computing the activation energies of late-transition-metal-catalyzed reactions with Au, Pt and Ir? *J. Chem. Theory Comput.* **2012**, *8*, 3119-3127.

[18] Neese, F. The ORCA program system. *WIREs Comput. Mol. Sci.* **2012**, *2*, 73-78.

[19] Klamt, A.; Schüürmann, G. COSMO: a new approach to dielectric screening in solvents with explicit expressions for the screening Energy and its gradients. *J. Chem. Soc., Perkin Trans. 2* **1993**, *0*, 799-805.

[20] Klamt, A. Conductor-like screening model for real solvents: a new approach to the quantitative calculation of salvation phenomena. *J. Phys. Chem.* **1995**, *99*, 2224-2235.

[21] Klamt, A.; Jonas, V. Treatment of the outlying charge in continuum solvation models. *J. Chem. Phys.* **1996**, *105*, 9972-9981.
